# Multi-model genome-wide association studies identify stable QTLs and candidate genes for lodging resistance in *indica* rice

**DOI:** 10.3389/fpls.2026.1914726

**Published:** 2026-09-18

**Authors:** Xiaoyun Luo, Jing Cao, Desuo Yin, Hualin Dong, Hongbo Wang, Jianlin Hu, Lian Xue, Peng Hu, Dan Liu, Yi Wen, Dongpan Chen, Jiabao Wang, Lanzhi Li, Deze Xu, Xingfei Zheng

**Affiliations:** 1 Institute of Food Crops, Hubei Academy of Agricultural Sciences, Wuhan, China; 2 College of Plant Protection, Hunan Agricultural University, Changsha, China; 3 Institute of Environment and Sustainable Development in Agriculture, Chinese Academy of Agricultural Sciences, Beijing, China

**Keywords:** candidate gene, culm breaking, GWAS, *indica* rice, lodging resistance

## Abstract

Lodging significantly limits rice yield and grain quality. Despite the identification of numerous QTLs and genes associated with lodging-related traits, the genetic framework for lodging resistance remains largely unclear. In this study, we evaluated 17 lodging-related traits over three consecutive years using a global collection of 370 *indica* rice accessions. From correlation analysis, eight representative traits, including plant height (PH), breaking resistance (BR), fresh weight (FW), stem length (SL), section modulus (SM), bending stress (BS), lodging index (LI), and the optimized parameter △BM, were selected for a genome-wide association study (GWAS). This study utilized 2,315,020 high-quality SNPs analyzed through the MLM, FarmCPU, and BLINK models. These models identified 10, 24, and 16 significant QTLs, respectively, with two stable QTLs, *qBS3* and *q△BM9*, consistently detected across all models. Haplotype analysis and expression profiling narrowed the candidate genes within the *qBS3* and *q△BM9* intervals down to seven, highlighting *LOC_Os03g49610* (encoding beta-glucosidase 8) and *LOC_Os09g28110* (encoding a hydroxyproline-rich glycoprotein) as promising candidate genes that warrant further functional validation for their roles in culm mechanical properties. Collectively, our results provide a valuable set of QTLs and candidate genes for marker-assisted breeding and offer mechanistic insights into the genetic basis of lodging resistance in *indica* rice, with the caveat that the two stable QTLs lie in close proximity to previously characterized loci and require fine-mapping to establish their independence.

## 1 Introduction

Rice (*Oryza sativa* L.) is the staple food for over half of the global population, playing a crucial role in worldwide food security. Unfortunately, lodging significantly reduces rice yields and deteriorates grain quality (Lang et al., 2012). During the “Green Revolution” of the 1960s, breeders improved plant lodging resistance by developing semi-dwarf rice varieties, which greatly increased global grain production and alleviated the food shortage crisis (Khush, 2001). However, the widespread adoption of high-yielding varieties, excessive use of chemical fertilizers, simplified cultivation techniques, and frequent extreme climate events have recently increased the risk of lodging in rice (Dalrymple, 1985; Wang et al., 2021b; Luo et al., 2022a; Wu et al., 2026). Consequently, improving lodging resistance in rice is now a critical challenge for maintaining global food security.

Lodging refers to the irreversible collapse of crops from their upright position to the ground (Mulder, 1954). There are three types of lodging in rice: culm bending, culm breaking, and root lodging (Hirano et al., 2017). This study specifically examines culm breaking type lodging. Various traits contribute to lodging resistance, such as plant height (PH) and fresh weight (FW), stem length (SL) of the basal internodes, plumpness of the leaf sheath, culm diameter (CD) and culm wall thickness (CT) of the basal internodes, and breaking resistance (BR) of the basal internodes (Islam et al., 2007; Zhang et al., 2014; Guo et al., 2021; Nomura et al., 2023). BR is indicated by its bending moment at breaking (M), which comprises two components: section modulus (SM), affected by CD and CT; and bending stress (BS), influenced by cell wall components such as cellulose and lignin (Ookawa and Ishihara, 1993, 1997; Ookawa et al., 2010). Typically, lodging resistance against culm breaking is assessed using the lodging index (LI) (Seko, 1962). To enhance the accuracy of lodging resistance evaluation, Luo et al. (2022b) proposed an optimized parameter, △BM, which quantifies the external force that the basal internodes can withstand, to complement the lodging index (LI).

To date, several genes related to lodging resistance in rice have been successfully cloned. The gene *SBI* encodes gibberellin 2-oxidase, which reduces gibberellin content, thereby shortening basal internodes and enhancing lodging resistance (Liu et al., 2018). *OsFBK4* and *OsUBR7* affect lodging resistance by regulating plant height (Zegeye et al., 2022; Zheng et al., 2022). *STRONG1* encodes a microtubule-associated protein MAP70, which negatively regulates culm diameter and lodging resistance (Zhao et al., 2025a). The R2R3-type MYB transcription factor, *RRS1*, negatively regulates culm mechanical strength by activating *STRONG1* expression (Gao et al., 2026). *STRONG2* increases culm diameter and cell wall components when overexpressed, and is directly activated by the transcription factor MYB61 (Zhao et al., 2025b). *OsRLCK191* and *OsDof1* improve lodging resistance by thickening the culm wall (Chang et al., 2024; Hu et al., 2026). A single-nucleotide substitution in *TC4*, was shown to cause a thin and brittle culm phenotype with weakened mechanical strength, indicating that *TC4* regulates culm development and mechanical strength through plant hormones and sugar transport (Zhou et al., 2025). The pleiotropic genes *SCM2* (*APO1*), *Gn1a* (*OsCKX2*), and *SCM3* (*FC1*) not only enhance lodging resistance by increasing culm diameter and wall thickness, but also boost grain yield by raising spikelet number per panicle (Ookawa et al., 2010; Yano et al., 2015; Tu et al., 2022). Other genes, including *OsWRKY102*, *OsBC17*, *OsPSLSq6*, *OsPEX1*, *OsTCP19*, and *OsXAT4*, influence lodging resistance by regulating stem hardness through the modulation of cell wall components (Ke et al., 2019; Zhao et al., 2021; Li et al., 2022a; Lv et al., 2024; Dai et al., 2026; Li et al., 2026; Lu et al., 2026; Mamidi et al., 2026). Furthermore, numerous QTLs associated with lodging-related traits of rice have been identified (Ookawa et al., 2016; Mulsanti et al., 2018; Sowadan et al., 2018; Kashiwagi, 2022; Bian et al., 2024; Gopi et al., 2026). These findings, along with the aforementioned genes, highlight the complex genetic regulation of lodging resistance in rice.

Despite recent advances, most identified genes and QTLs have been discovered using bi-parental mapping populations or mutant libraries, which often suffer from limited resolution and narrow genetic diversity. Additionally, the traditional map-based cloning strategy for genes and QTLs is extremely troublesome and time-consuming. However, with the rapid development of high-throughput sequencing technologies and bioinformatics, genome-wide association study (GWAS) has become a useful and robust strategy complementary to classical bi-parental mapping and is powerful for genetic dissection of complex traits (Huang et al., 2010; Zhao et al., 2011; Korte and Farlow, 2013; Huang and Han, 2014). In recent years, GWAS has been widely used to identify genes and QTLs associated with complex agronomic traits in rice (Yano et al., 2016; Zhang et al., 2019b; Wang et al., 2021a; Liu et al., 2023; Sachdeva et al., 2024). However, only a few studies have applied GWAS to explore lodging resistance in rice (Meng et al., 2021; Rashid et al., 2022b; Lu et al., 2026). In this study, we performed a multi-model GWAS (MLM, FarmCPU, and BLINK) on 17 lodging-related traits over three consecutive years in a diverse panel of 370 *indica* rice accessions. The study aimed to: (1) characterize the phenotypic variation and heritability of lodging-related traits in *indica* rice accessions; (2) identify stable QTLs associated with lodging resistance using multiple GWAS models; (3) evaluate high- confidence candidate genes within the stable QTLs through haplotype analysis, expression profiling, and functional annotation. The results will not only provide valuable genetic resources for improving lodging resistance in rice breeding programs, but also offer insights into the genetic mechanisms underlying lodging resistance in *indica* rice.

## 2 Materials and methods

### 2.1 Plant materials and field experiments

A natural population of 370 *indica* rice accessions distributed worldwide was used in this study (**Supplementary Table 1**). The field experiments were conducted at the experiment station of the Institute of Food Crops, Hubei Academy of Agriculture Sciences, Wuhan, China, during the growing seasons from May to October in 2023, 2024 and 2025. Following germination, seeds from each accession were cultivated in a seedling bed. At 25 days old, seedlings were manually transplanted into the experimental field. In each year, each accession was grown in one field plot consisting of 5 rows with 6 plants per row, giving 30 plants per plot. The hill spacing was 20 cm between rows and 16 cm between plants. Five individual plants were sampled from each plot for phenotypic measurements, and their arithmetic mean was used as the phenotypic value of that accession in that year. These five plants were subsamples within the same field experimental unit and were not treated as independent field replicates. Field management adhered to local standard practices.

### 2.2 Phenotyping of lodging-related traits

Lodging-related traits for each accession were determined 25 days after the full-heading date (more than 80% of panicles had emerged from the flag leaf sheath). To minimize edge effects, representative main tillers of 5 individual plants were picked from the middle of each plot for measuring the 17 lodging-related traits. Plant height (PH) was recorded from the ground to the top tip of the panicle. Culm length (CL) was measured from the lower node of the second basal internode to the top tip of the panicle. Stem length (SL) was determined from the lower node of the second basal internode to the lower node of the third basal internode. Fresh weight (FW) was calculated between the lower node of the second basal internode and the top tip of the panicle. Breaking resistance (BR) was assessed at the midpoint of the second basal internode with the leaf sheath, using a plant lodging tester (YYD-1, Zhejiang TOP Instrument Co.), following a previously reported method (Ookawa and Ishihara, 1992). Each sampled basal second internode with leaf sheath was placed on the groove of support pillars at a distance of 4 cm. The tester was aligned perpendicularly to the middle internode and progressively loaded until the internode broke, at which point the BR value (kg) was recorded. Additionally, the external and internal diameters of the minor and major axes of the oval cross-section at the center of the basal second internode were measured using a digital vernier caliper. Physical parameters were calculated as described by our previous studies (Luo et al., 2022a, 2022b) as follows:

Bending moment of the whole plant (BM, g·cm) = CL (cm) × FW (g)
Bending moment at breaking (M, g·cm) = 1/4 × [BR (kg) × 10^3^] × distance between supporting pillars (cm)
Lodging index (LI, %) = BM/M × 100%
The external force that the basal second internode can withstand (△BM, g·cm) = 2M – BM
Section modulus (SM, mm^3^) = π/32 × (a_1_^3^b_1_ − a_2_^3^b_2_)/a_1_
Bending stress (BS, g·mm^−2^) = (M × 10)/SM
Culm diameter (CD, mm) = (a_1_ + b_1_)/2
Culm wall thickness (CT, mm) = (a_1_ - a_2_ + b_1_ - b_2_)/4

where a_1_ and b_1_ respectively denote the outer diameters of the minor and major axes in an oval cross-section, whereas a_2_ and b_2_ respectively represent the inner diameters of the minor and major axes in an oval cross-section.

The best linear unbiased prediction (BLUP) values for lodging-related traits across three years were generated using the lme4 package in R 4.2.0 using the model lmer(Value ~ Year + (1 | Genotype)), with Year as a fixed factor (reference level 2023) and accession as a random effect; the genotypic value used for GWAS was BLUP_j = μ̂_2023 + û_j, where μ̂_2023 is the model intercept (the 2023 fixed-effect mean) and û_j is the best linear unbiased predictor of the random effect of accession j. Correlation analysis was conducted and visualized with the corrplot package in R 4.2.0. Phenotypic data were analyzed using SPSS version 20.0 (IBM, Armonk, NY, 2012). Broad-sense heritability (*H*^2^) for each trait across the three years was estimated as *H*² = σ²_g/(σ²_g + σ²_e/n_rep) following Knapp et al. (1985), with n_rep = 3. Because each accession had a single observation per year, the residual variance includes the genotype × year interaction, which could not be separated from residual error in this design.

### 2.3 Genotyping

The genotype data of the 370 *indica* rice accessions were obtained from the publicly available resequencing dataset deposited in NCBI BioProject PRJNA321462 and PRJNA331215 (2016) and described by Meng et al. (2021). The raw sequencing reads (average depth of approximately 11× per genome) were downloaded and further processed to eliminate adapter contamination and low-quality reads. The clean reads were then aligned to the Os-Nipponbare-Reference-IRGSP-1.0 using Burrows-Wheeler Aligner (BWA) (Li and Durbin, 2009). After alignment, single nucleotide polymorphism (SNP) calling was conducted using the Genome Analysis Toolkit (GATK) software (Van der Auwera et al., 2018). A stringent filtering strategy was applied to choose high-quality SNPs. Initially, heterozygous SNPs were considered missing, and IMPUTE2 was employed to impute these missing genotypes (Howie et al., 2009). Subsequently, SNPs with a minor allele frequency (MAF) < 0.05 were excluded. Additionally, SNPs with missing genotype rates > 0.2 were removed. Ultimately, a total of 2,315,020 high-quality SNPs were identified for further analysis.

### 2.4 Population structure and linkage disequilibrium analysis

Principal component analysis (PCA) and kinship estimation were utilized to elucidate the genetic structure of the population through the Genomic Association and Prediction Integrated Tool (GAPIT) version 3 within R 4.2.0 (Wang and Zhang, 2021). A distance matrix was calculated from all high-quality SNPs using SNPhylo (Lee et al., 2014), and an unweighted neighbor-joining tree was subsequently visualized by iTOL (https://itol.embl.de/). Linkage disequilibrium (LD) decay was assessed by calculating the mean squared correlation coefficient (*r^2^*) values pairwise within a 5 Mb range, employing the PopLDdecay software (Zhang et al., 2019a). When the mean value of *r^2^* decreased to half of its maximum value, the corresponding physical distance traveled by chromosome was taken as the LD decay distance (Huang et al., 2010; Du et al., 2022).

### 2.5 Genome-wide association study

The genome-wide association study (GWAS) was conducted with the R 4.2.0 software package GAPIT version 3 using the mixed linear model (MLM), fixed and random model circulating probability unification (FarmCPU) and Bayesian information and linkage disequilibrium iteratively nested keyway (BLINK) models (Yu et al., 2005; Liu et al., 2016; Huang et al., 2019). In all three GWAS models (MLM, FarmCPU, and BLINK), the first three principal components were fitted as fixed covariates to control for population structure, and the VanRaden kinship matrix was included as a random effect. To determine genome-wide significance, the Bonferroni multiple test correction was applied, setting the *p* value threshold at 0.05/n, where n represents the number of SNPs. Since the number of SNPs was 2,315,020, the SNP-trait associations were declared significant when the *p* value < 2.16e-8 or -log_10_ (*p*) > 7.67, and the SNP with the minimum *p* value was considered as the peak SNP. Manhattan and quantile-quantile (Q-Q) plots were generated using the GAPIT version 3 package in R 4.2.0.

### 2.6 Candidate genes identification and haplotype analysis

The QTL regions were defined by the LD decay distance left and right genomic regions of significant SNPs (SNP ± LD decay distance). Overlapping adjacent regions were merged into a single QTL (Meng et al., 2021; Li et al., 2022b). A QTL that met any of the following criteria was considered as an important QTL: (1) repeatedly detected over at least two years; (2) identified within BLUP; (3) consistently found in at least two models; (4) influenced multiple traits; (5) located near a reported cloned gene or a fine-mapped QTL. Candidate genes for the important QTLs were identified via the following steps. First, all genes within each important QTL were retrieved from the Rice Genome Annotation Project (https://rice.uga.edu/). LD block analysis was performed around the peak SNP of each important QTL using pairwise *r*² calculated with PLINK v1.9, to evaluate whether the candidate region could be narrowed on the basis of local LD. Next, non-synonymous SNPs in the coding regions of these genes were extracted for haplotype analysis using the geneHapR package in R 4.2.0 (Zhang et al., 2023), excluding those annotated as expressed protein, hypothetical protein, transposon protein, and retrotransposon protein. These annotations were excluded due to their poorly characterized functions or lack of direct association with structural or metabolic pathways affecting culm mechanical properties, thereby narrowing the focus to genes with established or plausible biological relevance to lodging resistance. It should be noted that the suffix “, expressed” attached to a functional description is an expression-evidence qualifier in the Rice Genome Annotation Project and does not correspond to the expressed protein category. Haplotypes with fewer than 10 accessions were discarded. Subsequently, a Student’s t-test was performed to examine significant phenotypic variations among the haplotypes. Finally, genes exhibiting significant differences among haplotypes for each important QTL were identified as candidate genes. In addition, SNPs in the promoter (2 kb upstream of the transcription start site) regions of the candidate genes were extracted and subjected to the same haplotype-phenotype association analysis, to evaluate regulatory natural variation.

## 3 Results

### 3.1 Population structure and linkage disequilibrium

After stringent filtering of the genomic data from 370 *indica* rice accessions, a total of 2,315,020 high-quality SNPs were detected to be distributed across all 12 chromosomes. The size of chromosome varied from 23.0 Mb for chromosome 9 to 43.3 Mb for chromosome 1. The number of SNPs on each chromosome ranged from 149,364 on chromosome 12 to 290,029 on chromosome 1. On average, the SNP distance across all chromosomes was 0.161 kb, with distances varying from 0.140 kb on chromosome 8 to 0.224 kb on chromosome 4. These SNPs covered 373,245,519 bp of the whole rice genome resulting in an average SNP density of 6.20 SNPs per kb (**Supplementary Table 2**).

All high-quality SNPs were used to estimate the principal components, kinship matrix, neighbor-joining tree, and linkage disequilibrium (LD). The score plot of the first two principal components, accounting for 54.67% of the variation, revealed that most accessions formed a continuous main cluster, whereas a small group of 41 accessions was separated from the main cluster along PC1 (**Figure 1A**). Consistent with this, the kinship heatmap showed a block of elevated pairwise relatedness corresponding to this group, together with only localized regions of moderate relatedness elsewhere (**Figure 1B**), and the neighbor-joining tree resolved a distinct clade comprising most of these accessions (28 of 41) (**Figure 1C**). When the *r^2^* decayed to half of the maximum value (0.65), the corresponding physical distance of LD decay was 92,954 bp (**Figure 1D**). The above results suggested that no strong or obvious population stratification was observed among the *indica* rice accessions, based on the PCA, kinship heatmap, and neighbor-joining analyses, which was beneficial for subsequent GWAS.

**Figure 1:**
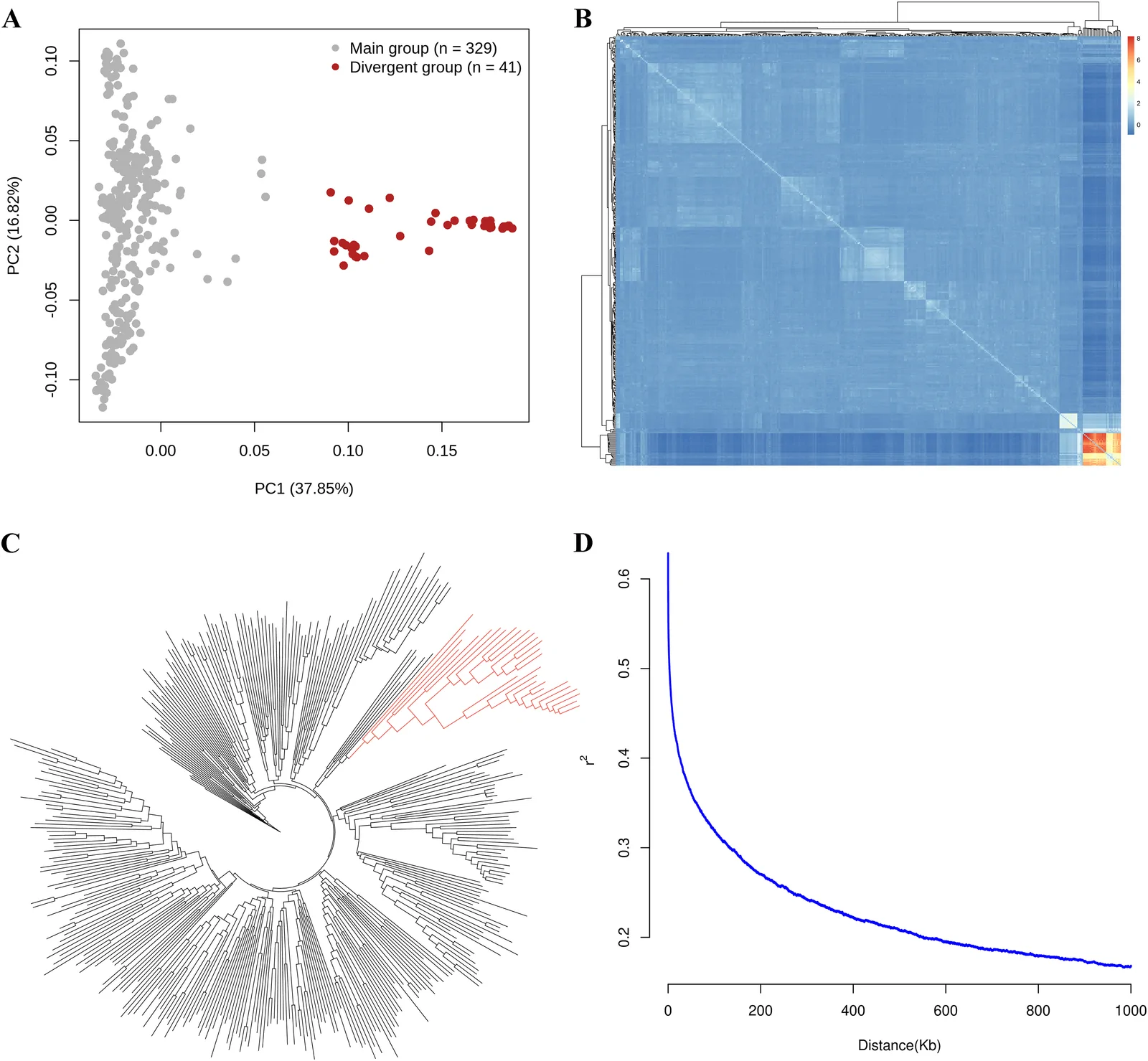
Population structure and LD decay of 370 *indica* rice accessions. **(A)** Principal component analysis. Accessions are colored according to the kinship-based groups with the small divergent group shown in red. PC1, first principal component; PC2, second principal component. Values in parentheses indicate percentage of variance explained by each principal component. **(B)** Heatmap of kinship with the tree shown on the top and left. **(C)** Neighbor-joining tree. The distinct clade comprising the 28 core accessions of the divergent group is highlighted in red. **(D)** LD decay analysis. Y axis is the average *r^2^* value of each 5 kb region and X axis is physical distance between markers.

### 3.2 Phenotypic variation and trait correlation

We measured 17 lodging-related traits in 370 *indica* rice accessions over three consecutive years. The correlation analysis results are presented in **Figure 2**. The correlations among lodging-related traits were similar in 2023, 2024, 2025, and the best linear unbiased prediction (BLUP). Notably, plant height (PH) exhibited a strong correlation with culm length (CL). Similarly, fresh weight (FW) was strongly correlated with the bending moment of the whole plant (BM). The correlation coefficient between breaking resistance (BR) and the bending moment at breaking (M) was 1.00, as per the calculation formula. The section modulus (SM) was strongly positively correlated with a_1_, b_1_, a_2_ and b_2_, with coefficients exceeding 0.84. Additionally, SM showed positive correlations with culm diameter (CD) and culm wall thickness (CT), with coefficients ranging from 0.88 to 0.97. These high correlations suggest that the traits are likely controlled by the same genetic factors. To reduce redundant results, we selected 8 traits (PH, BR, FW, SL, SM, BS, LI and △BM) from the 17 lodging-related traits for further analysis.

**Figure 2:**
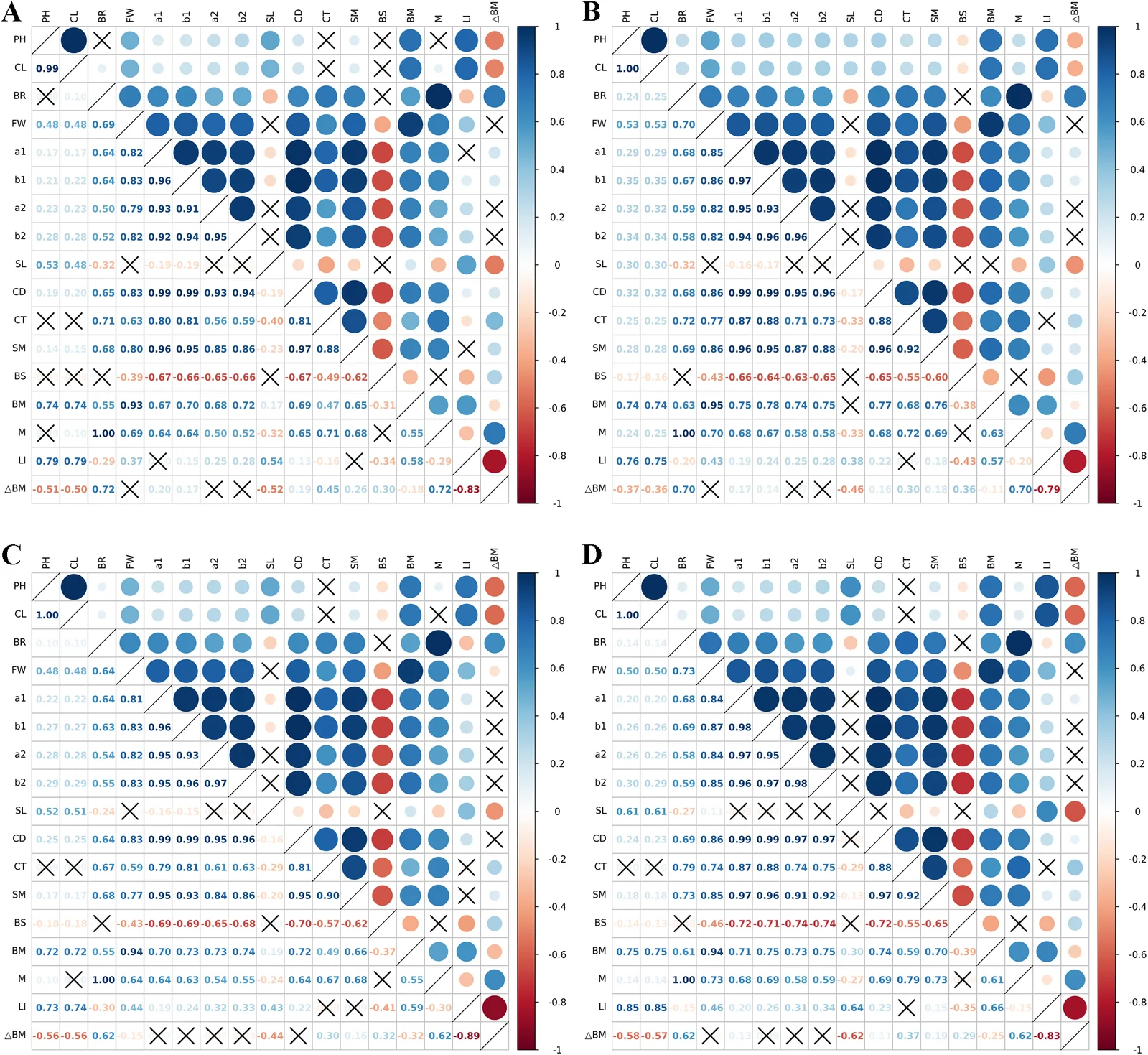
Correlation coefficients between 17 lodging-related traits in 2023 **(A)**, 2024 **(B)**, 2025 **(C)** and BLUP **(D)**. In the upper triangular portion, the size and color of each circle represent the magnitude of corresponding correlation coefficient. The lower triangular section displays the actual correlation coefficient values between the traits. The × denotes non-significant correlation at the 0.05 level.

The eight selected lodging-related traits showed significant variation among 370 *indica* rice accessions across the years 2023, 2024, 2025, and in the BLUP, with coefficients of variation (CV) ranging from 13.18% to 82.65% (**Table 1**). The broad-sense heritability (*H^2^*) for these traits was high ranging from 0.70 for SL to 0.94 for PH (**Table 1**), indicating that genetic factors were the primary contributors to variation in lodging resistance. An identical and slightly skewed frequency distribution was observed for all eight traits over the three years and in the BLUP (**Table 1**; **Figure 3**), suggesting their suitability for subsequent genome-wide association studies (GWAS).

**Table 1:** Descriptive statistics of the 8 selected lodging-related traits of 370 *indica* rice accessions in 2023, 2024, 2025, and BLUP.

Trait	Year	Minimum	Maximum	Mean	SD	CV (%)	Skewness	Kurtosis	*H^2^*
PH (cm)	2023	82.2	167.9	111.1	16.4	14.75	1.12	1.44	0.94
	2024	60.7	164.8	107.7	17.0	15.79	0.37	0.22	
	2025	75.2	168.3	113.2	15.9	14.02	0.83	1.42	
	BLUP	80.6	162.5	111.1	14.6	13.18	0.86	1.07	
BR (kg)	2023	0.43	3.60	1.35	0.42	31.38	0.66	2.05	0.78
	2024	0.48	3.84	1.22	0.44	35.77	1.18	3.37	
	2025	0.53	3.46	1.21	0.36	29.86	1.24	4.58	
	BLUP	0.82	3.00	1.35	0.26	19.63	1.29	4.67	
FW (g)	2023	6.67	31.24	15.05	4.33	28.78	0.70	0.84	0.89
	2024	4.45	35.86	14.03	4.64	33.06	1.09	1.91	
	2025	6.58	32.99	15.38	4.26	27.68	0.75	1.26	
	BLUP	8.31	29.60	15.05	3.54	23.51	0.71	0.71	
SL (cm)	2023	5.8	24.1	12.1	3.4	28.08	0.84	0.63	0.70
	2024	4.5	30.5	12.1	3.5	29.23	0.73	1.76	
	2025	4.7	25.0	12.0	3.6	30.09	1.05	1.09	
	BLUP	8.6	19.1	12.1	1.9	16.02	1.06	1.28	
SM (mm^3^)	2023	5.29	71.76	22.87	10.14	44.33	1.08	1.83	0.80
	2024	2.93	77.81	20.61	10.52	51.06	1.49	3.24	
	2025	4.24	63.16	20.46	9.12	44.58	1.36	2.73	
	BLUP	10.95	45.67	22.87	6.73	29.44	1.01	0.92	
BS (g·mm^−2^)	2023	273.80	1612.17	646.92	210.99	32.61	1.19	2.69	0.74
	2024	224.03	1718.80	664.95	230.14	34.61	1.30	3.15	
	2025	285.76	2247.93	657.44	228.01	34.68	1.82	7.16	
	BLUP	373.27	1360.09	646.92	134.06	20.72	1.42	3.89	
LI (%)	2023	53.19	321.74	118.51	41.47	35.00	1.50	4.07	0.81
	2024	38.54	284.43	120.82	42.95	35.55	0.98	1.15	
	2025	44.25	322.50	140.45	46.14	32.85	0.73	0.78	
	BLUP	54.02	225.22	118.51	30.14	25.43	0.80	0.77	
△BM (g·cm)	2023	-1323.19	4316.51	1149.25	716.03	62.30	0.38	1.48	0.75
	2024	-702.24	4220.59	1007.81	682.66	67.74	0.83	2.64	
	2025	-926.84	4064.15	774.83	640.41	82.65	0.47	1.86	
	BLUP	-88.46	3093.05	1149.25	417.99	36.37	0.58	2.20	

PH, plant height; BR, breaking resistance; FW, fresh weight; SL, stem length; SM, section modulus; BS, bending stress; LI, lodging index; △BM, external force that the basal second internode could withstand; SD, standard deviation; CV, coefﬁcient of variance; *H^2^*, broad-sense heritability.

**Figure 3:**
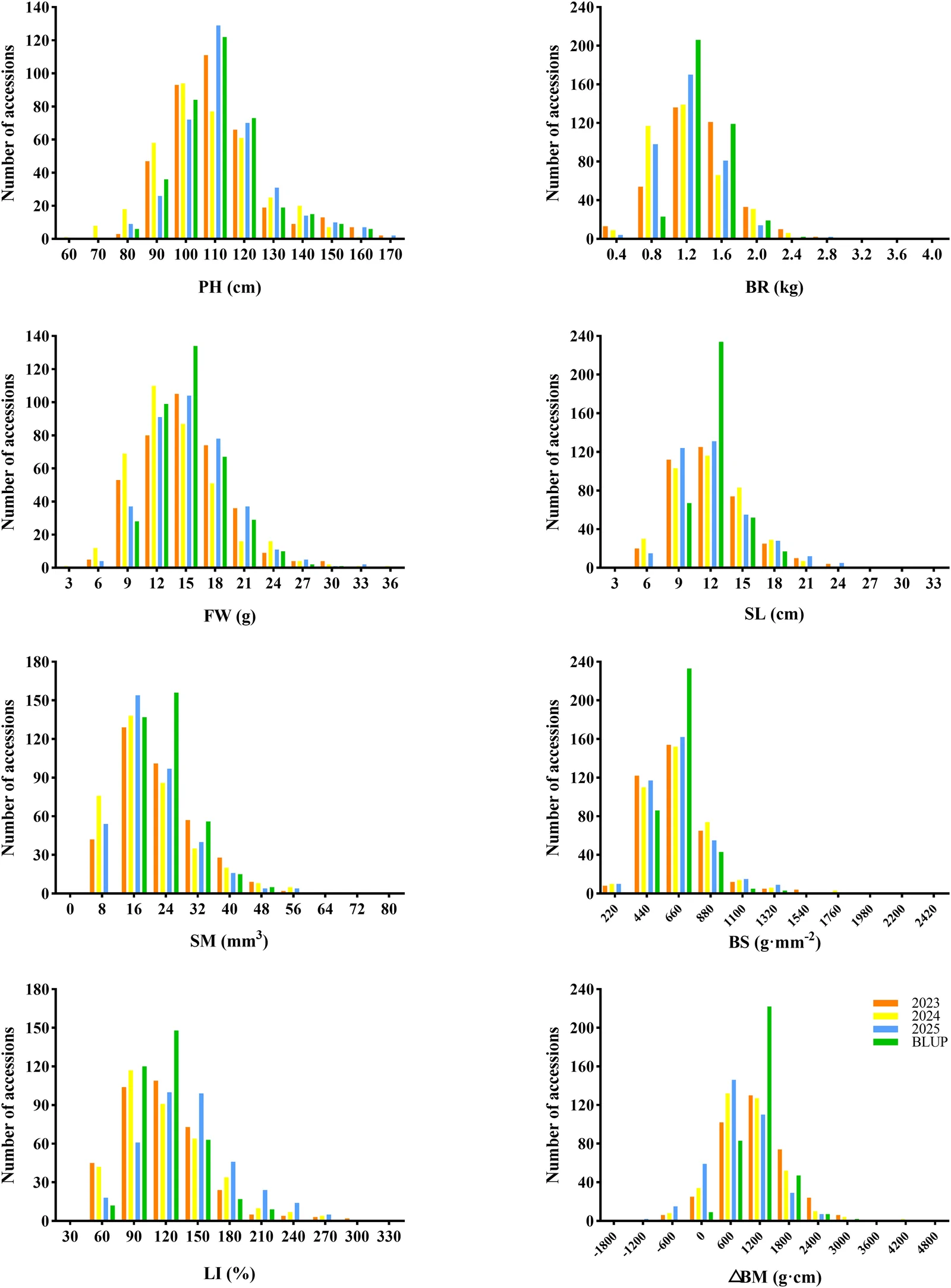
Distributions of the selected lodging-related traits (PH, BR, FW, SL, SM, BS, LI, and ΔBM) of 370 *indica* rice accessions in 3 years and BLUP. PH, plant height; BR, breaking resistance; FW, fresh weight; SL, stem length; SM, section modulus; BS, bending stress; LI, lodging index; △BM, external force that the basal second internode could withstand.

### 3.3 Multi-model GWAS and QTL analysis

The multi-model GWAS was conducted to detect the significant SNPs associated with the eight selected lodging-related traits among the 370 *indica* rice accessions in 2023, 2024, 2025, and BLUP. Using the MLM, 47 significant SNPs for the investigated traits except for FW and LI were discovered on chromosomes 1, 2, 3, 7, 9, and 12 (**Figure 4**; **Supplementary Figure 1**; **Supplementary Table 3**). The FarmCPU model yielded 54 significant SNPs associated with the target traits except for BR, which were distributed across all chromosomes except chromosome 12 (**Supplementary Figure 2**; **Supplementary Table 3**). Meanwhile, the BLINK model identified 30 significant SNPs for the selected traits excluding SM. These SNPs were located on all chromosomes except 4 and 6 (**Supplementary Figure 3**; **Supplementary Table 3**). The Q-Q plots for all traits demonstrated effective control of false positive associations, with observed *p*-values closely following the expected null distribution and showing no evidence of global inflation (**Supplementary Figures 4**–**6**). This calibration supports the reliability of the significant SNP-trait associations identified by the multi-model GWAS.

**Figure 4:**
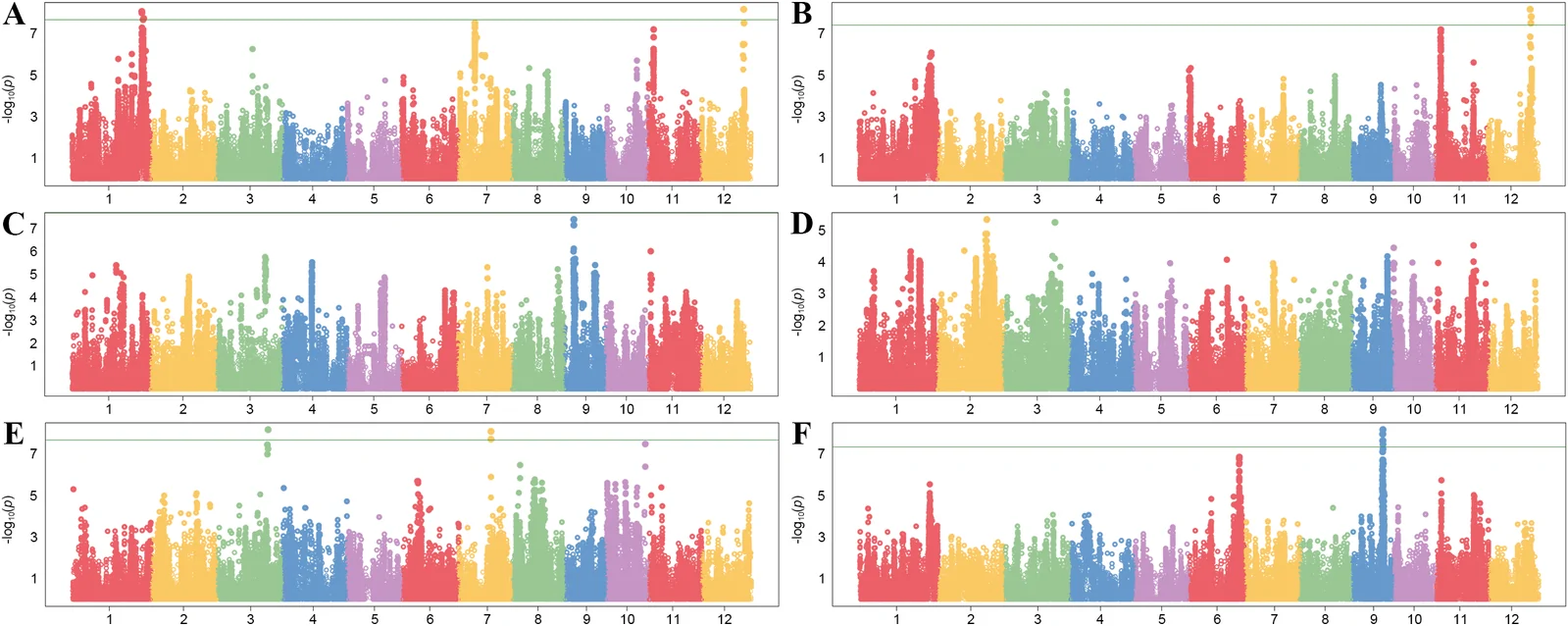
Manhattan plots of the GWAS results for the 8 selected lodging-related traits with MLM in BLUP. **(A)** PH; **(B)** SL; **(C)** BR; **(D)** SM; **(E)** BS; **(F)** △BM. The green horizontal line represents the genome-wide significance threshold of Bonferroni adjusted *p* value.

By the criteria of important QTL, all the above significant SNPs were delineated into 10, 24, and 16 QTLs with the MLM, FarmCPU, and BLINK models, respectively (**Table 2**; **Figure 5**). For PH, four QTLs were detected in the MLM, namely, *qPH1.1*, *qPH1.2*, *qPH1.3*, and *qPH12*; four QTLs were identified in the FarmCPU model, namely, *qPH1.2*, *qPH3.1*, *qPH3.2*, and *qPH4*; and only one QTL *qPH11* was observed in the BLINK model. Notably, *qPH1.2* was common to both the MLM and FarmCPU models. For SL, the MLM detected two QTLs, *qSL12.1* and *qSL12.2*. The FarmCPU model identified one QTL *qSL3*, while the BLINK model found one QTL *qSL10*. For FW, six QTLs were identified in the FarmCPU including *qFW2.1*, *qFW2.2*, *qFW5*, *qFW6*, *qFW8* and *qFW9.2*. Six QTLs were detected in the BLINK model including *qFW2.1*, *qFW5*, *qFW8*, *qFW9.1*, *qFW9.2* and *qFW11*. Four QTL were commonly identified in both models including *qFW2.1*, *qFW5*, *qFW8* and *qFW9.2*. For BR, the MLM identified one QTL *qBR9*, while the BLINK model detected two QTLs, *qBR2* and *qBR9*. Obviously, *qBR9* was observed in both models. Four QTLs (*qSM1*, *qSM3*, *qSM6* and *qSM9*) for SM were identified only in the FarmCPU model. For BS, two (*qBS3* and *qBS7.1*), two (*qBS3* and *qBS7.2*), and one (*qBS3*) QTLs were detected using the MLM, FarmCPU and BLINK models, respectively. It was worth noting that *qBS3* was consistently found across all three models. For LI, *qLI4.1*, *qLI4.2*, and *qLI10* were identified with the FarmCPU model, whereas *qLI11* and *qLI12* were detected with the BLINK model. Of the △BM associated QTLs, 1, 4, and 3 QTLs were separately identified by the MLM, FarmCPU and BLINK models. Among these QTLs, *q△BM7* and *q△BM11* were common across the FarmCPU and BLINK models, while *q△BM9* was observed in all three models.

**Table 2:** Important QTLs for the 8 selected lodging-related traits identified by multi-model GWAS in 2023, 2024, 2025, and BLUP.

Model	Trait	QTL	Year	Chromosome	Start position	End position	Peak SNP	MAF	*P* value	FDR
MLM	PH	*qPH1.1*	2023	1	38,380,032	38,610,348	38,472,986	0.165	8.21E-09	0.00317
			BLUP	1	38,380,032	38,610,348	38,472,986	0.165	8.65E-09	0.00303
		*qPH1.2*	2024	1	39,078,261	39,264,169	39,171,215	0.308	2.80E-09	0.00564
			BLUP	1	39,078,261	39,264,169	39,171,215	0.308	2.13E-08	0.00303
		*qPH1.3*	BLUP	1	39,425,298	39,611,206	39,518,252	0.078	1.86E-08	0.00303
		*qPH12*	2023	12	23,471,088	23,828,904	23,564,042	0.059	7.31E-10	0.00129
			2025	12	23,471,088	23,656,996	23,564,042	0.059	8.18E-09	0.00163
			BLUP	12	23,471,088	23,656,996	23,564,042	0.059	6.82E-09	0.00303
	SL	*qSL12.1*	2025	12	23,072,766	23,274,660	23,165,720	0.070	2.18E-11	0.00005
			BLUP	12	23,072,766	23,258,674	23,165,720	0.070	3.55E-09	0.00165
		*qSL12.2*	2025	12	23,471,088	23,828,904	23,721,929	0.059	1.28E-09	0.00099
			BLUP	12	23,471,088	23,828,904	23,721,929	0.059	8.19E-09	0.00165
	BR	*qBR9*	2024	9	5,289,816	5,475,788	5,382,770	0.070	1.44E-08	0.01673
	BS	*qBS3*	BLUP	3	28,231,368	28,417,276	28,324,322	0.051	6.90E-09	0.00967
		*qBS7.1*	BLUP	7	17,535,498	17,727,224	17,628,452	0.051	8.35E-09	0.00967
	△BM	*q△BM9*	2024	9	16,966,287	17,152,195	17,059,241	0.078	1.72E-08	0.00257
			BLUP	9	16,818,097	17,182,941	17,059,241	0.078	2.91E-09	0.00084
FarmCPU	PH	*qPH1.2*	2024	1	39,078,261	39,264,169	39,171,215	0.308	2.53E-13	5.86E-07
			BLUP	1	39,078,261	39,264,169	39,171,215	0.308	2.14E-11	0.00005
		*qPH3.1*	2024	3	20,022,926	20,208,834	20,115,880	0.114	1.81E-09	0.00084
		*qPH3.2*	BLUP	3	22,914,907	23,100,815	23,007,861	0.119	1.11E-08	0.01288
		*qPH4*	2023	4	4,683,464	4,869,372	4,776,418	0.192	1.97E-08	0.01518
			2024	4	4,683,278	4,869,186	4,776,232	0.332	9.33E-11	0.00007
	SL	*qSL3*	2023	3	20,022,926	20,208,834	20,115,880	0.114	2.74E-09	0.00635
	FW	*qFW2.1*	2025	2	30,266,947	30,452,855	30,359,901	0.430	1.87E-15	4.33E-09
		*qFW2.2*	BLUP	2	20,021,378	20,207,286	20,114,332	0.270	6.97E-09	0.00806
		*qFW5*	2025	5	18,584,551	18,770,459	18,677,505	0.068	1.33E-14	1.53E-08
			BLUP	5	18,584,551	18,770,459	18,677,505	0.068	1.86E-10	0.00043
		*qFW6*	2024	6	30,068,697	30,254,605	30,161,651	0.054	2.33E-11	0.00003
		*qFW8*	2025	8	3,864,072	4,049,980	3,957,026	0.073	1.16E-10	0.00007
		*qFW9.2*	2025	9	19,914,075	20,099,983	20,007,029	0.289	4.94E-11	0.00004
	SM	*qSM1*	BLUP	1	33,235,798	33,421,706	33,328,752	0.143	1.38E-08	0.01593
		*qSM3*	BLUP	3	28,457,865	28,643,773	28,550,819	0.054	6.07E-10	0.00140
		*qSM6*	2024	6	30,068,697	30,254,605	30,161,651	0.054	1.12E-09	0.00259
		*qSM9*	2023	9	17,132,552	17,318,460	17,225,506	0.124	1.41E-08	0.01636
	BS	*qBS3*	2023	3	28,231,368	28,417,276	28,324,322	0.051	1.06E-08	0.01222
			BLUP	3	28,231,368	28,417,276	28,324,322	0.051	2.14E-11	0.00005
		*qBS7.2*	BLUP	7	26,038,424	26,224,332	26,131,378	0.303	6.60E-09	0.00764
	LI	*qLI4.1*	BLUP	4	23,512,643	23,698,551	23,605,597	0.054	1.52E-09	0.00183
		*qLI4.2*	BLUP	4	30,102,229	30,288,137	30,195,183	0.214	1.58E-09	0.00183
		*qLI10*	BLUP	10	17,442,790	17,628,698	17,535,744	0.078	6.87E-09	0.00530
	△BM	*q△BM7*	2025	7	28,288,731	28,474,639	28,381,685	0.076	1.16E-09	0.00120
		*q△BM8*	BLUP	8	19,325,533	19,511,441	19,418,487	0.097	6.01E-09	0.00463
		*q△BM9*	2023	9	16,818,097	17,004,005	16,911,051	0.076	9.83E-15	2.28E-08
			2024	9	16,818,097	17,004,005	16,911,051	0.076	6.59E-13	1.52E-06
			BLUP	9	16,818,097	17,004,005	16,911,051	0.076	3.27E-18	7.56E-12
		*q△BM11*	2025	11	2,981,520	3,167,428	3,074,474	0.103	7.74E-11	0.00018
			BLUP	11	2,981,520	3,167,428	3,074,474	0.103	3.88E-15	4.49E-09
BLINK	PH	*qPH11*	2024	11	2,998,085	3,183,993	3,091,039	0.103	3.38E-10	0.00078
	SL	*qSL10*	BLUP	10	13,459,779	13,645,687	13,552,733	0.238	5.44E-09	0.01259
	FW	*qFW2.1*	2025	2	30,266,947	30,452,855	30,359,901	0.430	5.01E-11	0.00012
			BLUP	2	30,319,875	30,505,783	30,412,829	0.441	2.08E-08	0.01607
		*qFW5*	2025	5	18,584,551	18,770,459	18,677,505	0.068	5.22E-09	0.00403
		*qFW8*	2025	8	3,864,072	4,049,980	3,957,026	0.073	1.20E-08	0.00692
		*qFW9.1*	2024	9	11,541,639	11,727,547	11,634,593	0.281	5.70E-09	0.01319
			BLUP	9	11,541,639	11,727,547	11,634,593	0.281	1.64E-08	0.01607
		*qFW9.2*	2025	9	19,914,127	20,100,035	20,007,081	0.292	2.38E-09	0.00275
		*qFW11*	BLUP	11	20,240,132	20,426,040	20,333,086	0.135	1.96E-10	0.00045
	BR	*qBR2*	2024	2	19,955,277	20,141,185	20,048,231	0.400	5.59E-10	0.00129
			BLUP	2	19,951,319	20,137,227	20,044,273	0.397	3.06E-10	0.00071
		*qBR9*	2024	9	5,289,816	5,475,788	5,382,770	0.070	7.34E-09	0.00566
			BLUP	9	5,289,816	5,475,788	5,382,770	0.070	1.67E-08	0.00879
	BS	*qBS3*	2025	3	28,231,368	28,417,276	28,324,322	0.051	1.10E-08	0.02546
	LI	*qLI11*	2025	11	2,981,520	3,167,428	3,074,474	0.103	6.88E-11	0.00016
			BLUP	11	2,981,520	3,167,428	3,074,474	0.103	3.17E-09	0.00733
		*qLI12*	BLUP	12	23,929,236	24,115,144	24,022,190	0.143	1.34E-08	0.01549
	△BM	*q△BM7*	2025	7	28,288,731	28,474,639	28,381,685	0.076	1.22E-09	0.00283
		*q△BM9*	2023	9	16,818,097	17,004,005	16,911,051	0.076	4.45E-15	1.03E-08
			2024	9	16,818,097	17,004,005	16,911,051	0.076	7.14E-13	1.65E-06
			BLUP	9	16,818,097	17,004,005	16,911,051	0.076	9.96E-17	2.30E-10
		*q△BM11*	BLUP	11	2,981,520	3,167,428	3,074,474	0.103	6.55E-11	0.00008

MAF, minor allele frequency; FDR, false discovery rate.

**Figure 5:**
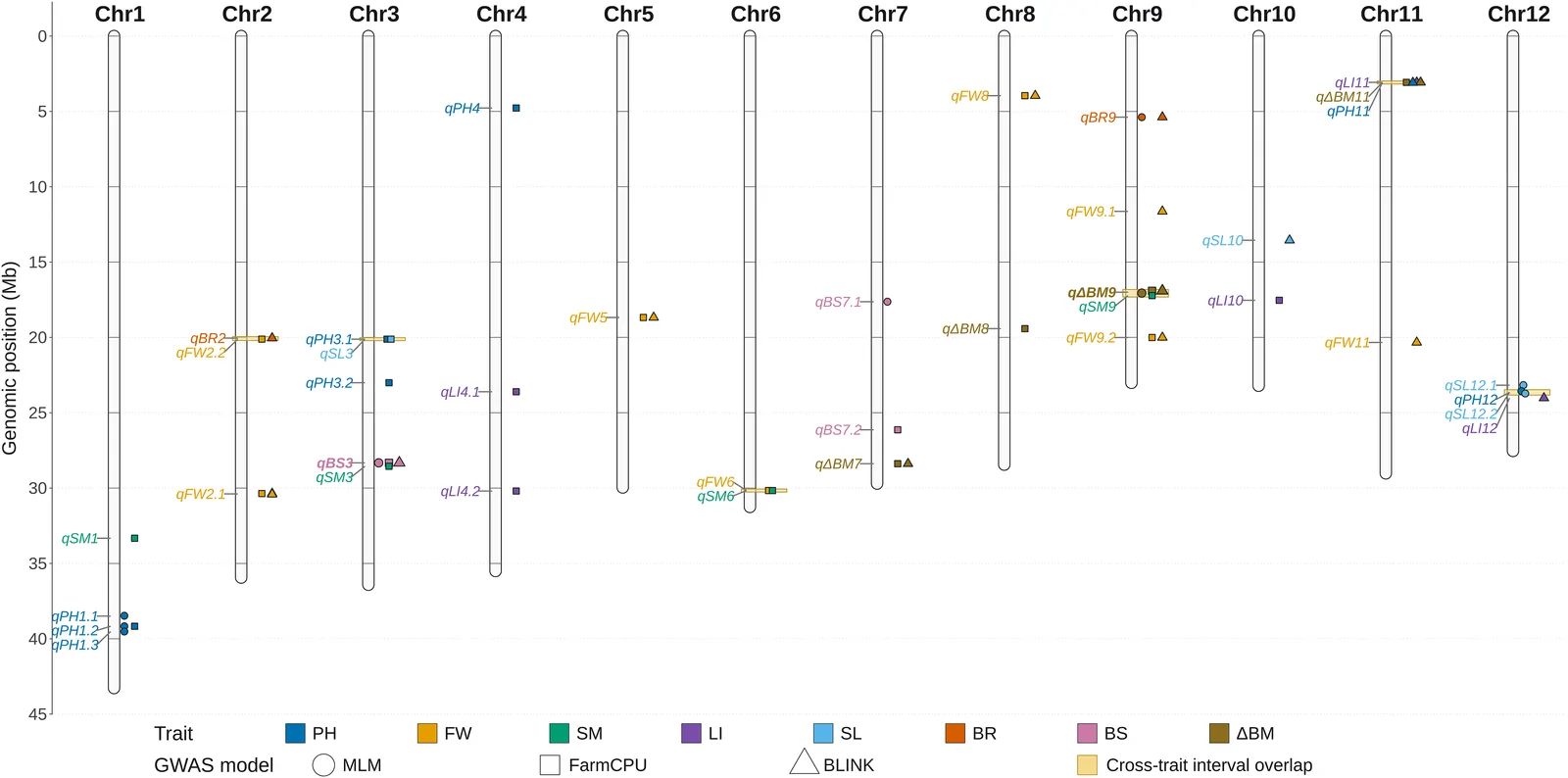
Genomic distribution of the important QTLs across the 12 rice chromosomes. Chromosomes are drawn to scale; QTL intervals are shown as colored bars according to trait, with separate tracks for the three GWAS models (MLM, FarmCPU, and BLINK) and the peak SNP of each QTL marked by the corresponding model symbol. Gold bands denote intervals shared by QTLs of different traits (co-localization).

The *qPH12* and *qSL12.2* in the MLM shared the same genomic region, hereafter, these two QTLs were regarded as a single QTL. Similarly, *qPH3.1* and *qSL3* were considered identical in the FarmCPU model. Additionally, *qFW6* and *qSM6* were also treated as the same QTL. In the BLINK model, *qPH11*, *qLI11* and *q△BM11* were found in overlapping adjacent regions, which were combined into the same QTL.

Among all the above important QTLs, two stable QTLs (*qBS3* and *q△BM9*) for BS and △BM were consistently identified in all three models, showed temporal stability across the three years and BLUP, and represented traits directly related to culm mechanical strength. For the two stable QTLs, the additive effect and the variance explained (△R²) of the peak SNP are reported for each model detection (**Supplementary Table 4**). The *qBS3* peak SNP showed an additive effect of 87.13 g·mm^-2^ and explained 6.95% of the phenotypic variance in MLM-BLUP, and the *q△BM9* peak SNP showed an additive effect of 474.24 g·cm with △R² = 7.51% in MLM-BLUP and of 743.85 g·cm with △R² = 7.86% in MLM-2024. This cross-model consistency and temporal stability substantially reduced the likelihood of false positives and indicated robust genetic effects. Therefore, further studies were conducted on these two stable QTLs to prioritise candidate gene discovery.

### 3.4 Identification of candidate genes for *qBS3*

There were 14 genes in *qBS3* in the range of 28.23-28.41 Mb on chromosome 3 after removal of genes encoding expressed proteins, hypothetical proteins, transposon proteins and retrotransposon proteins (**Supplementary Table 5**). Among these, the genes *LOC_Os03g49570*, *LOC_Os03g49610*, *LOC_Os03g49710*, and *LOC_Os03g49730* were found to contain non-synonymous SNPs (**Supplementary Table 7**). LD block analysis showed that the peak SNP of *qBS3* had no strong-LD partner within the interval (maximum pairwise *r*² = 0.39; no SNP with *r*² ≥ 0.8), and therefore no LD block could be delimited around it (**Supplementary Table 8**; **Supplementary Figure 7A**). Consequently, the candidate region could not be narrowed by LD in this panel, and all four genes in the interval were retained for the expression and haplotype analyses below. These four genes all exhibited relatively high expression levels in stems using the RNA-seq data published by the Rice Genome Annotation Project (**Figure 6A**). Each of the four genes contained two haplotypes, and they all showed significant differences in BS in BLUP between haplotypes (**Figures 6B–E**, **Supplementary Tables 9**, **11**), indicating that they were the candidate genes for *qBS3*. In addition, promoter haplotypes of the four candidate genes also differed significantly in BS in BLUP (**Supplementary Table 12**), providing complementary evidence of regulatory natural variation. It is noteworthy that the four genes were partitioned into two near-identical haplotype groups (316/51 and 309/58), consistent with the strong LD among their non-synonymous SNPs (*r²* = 0.820-1.000) (**Supplementary Table 13**). These genes therefore form a closely linked haplotype block, and the causal gene within this block cannot be distinguished by haplotype analysis alone. Moreover, the LD between the GWAS peak SNP of *qBS3* (chr03: 28,324,322; MAF 0.051) and these non-synonymous variants was low (*r*² = 0.003-0.010), reflecting the large difference in minor allele frequencies (**Supplementary Table 14**). The candidate genes were therefore prioritised on the basis of their own haplotype-trait associations, stem expression, and functional annotation rather than by LD with the peak SNP.

**Figure 6:**
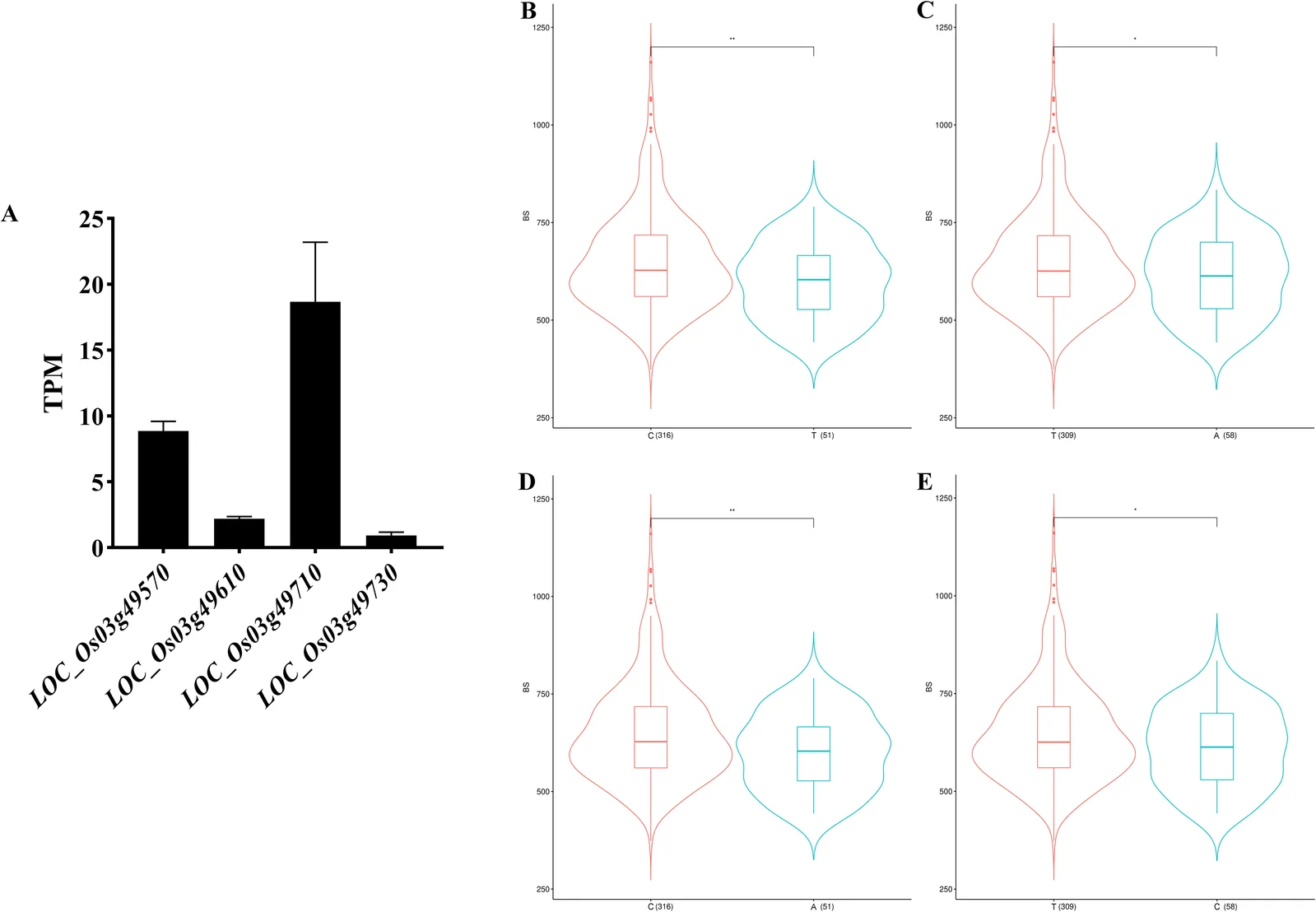
Stem expression levels of the four candidate genes based on the RNA-seq data of the Rice Genome Annotation Project **(A)** and haplotype analysis of the candidate genes *LOC_Os03g49570* **(B)**, *LOC_Os03g49610* **(C)**, *LOC_Os03g49710* **(D)** and *LOC_Os03g49730* **(E)** in *qBS3* with BLUP. The x-axis labels indicate the SNP composition and haplotype counts. The y-axis shows the BS values (g·mm^-2^). ^*^ *p* < 0.05, ^**^ *p* < 0.01 based on Student’s t-test.

### 3.5 Identification of candidate genes for *q△BM9*

For *q△BM9* in the 16.81-17.18 Mb region on chromosome 9, a total of 36 genes were found after excluding the expressed protein, hypothetical protein, transposon protein and retrotransposon protein (**Supplementary Table 6**). Among these, 11 genes exhibited non-synonymous mutations (**Supplementary Table 7**). LD block analysis showed that the two peak SNPs of *q△BM9* lie within a single large LD block (*r*² ≥ 0.8) spanning chr9: 16,829,251-17,249,775, which covers essentially the entire QTL interval (**Supplementary Table 8**; **Supplementary Figure 7B**). Therefore, the candidate region could not be narrowed by LD in this panel, and all 11 genes in the interval were retained for the expression and haplotype analyses. Based on RNA-seq data from the Rice Genome Annotation Project (https://rice.uga.edu/), genes with no or low expression levels in stems were excluded. The remaining genes, *LOC_Os09g27830*, *LOC_Os09g27900*, *LOC_Os09g28110*, and *LOC_Os09g28180* showed relatively high expression levels (**Figure 7A**). Haplotype analysis revealed significant differences for △BM in BLUP between different haplotypes for all genes except *LOC_Os09g28180* (**Figures 7B–E**; **Supplementary Tables 10**, **11**). Consequently, *LOC_Os09g27830*, *LOC_Os09g27900* and *LOC_Os09g28110* were considered as candidate genes for *q△BM9*. In addition, promoter haplotypes of the three candidate genes also differed significantly in △BM in BLUP (**Supplementary Table 12**), providing complementary evidence of regulatory natural variation. Similarly, the three candidate genes were partitioned into nearly identical haplotype groups (332/37, 331/38, and 326/43), reflecting the strong LD among their non-synonymous SNPs (*r²* = 0.819-0.971) (**Supplementary Table 13**). Consequently, these genes constitute a closely linked haplotype block whose individual members cannot be resolved by haplotype data alone. Furthermore, the LD between the GWAS peak SNPs of *q△BM9* (chr09: 16,911,051 and chr09: 17,059,241; MAF 0.076-0.078) and these non-synonymous variants was low (*r*² = 0.009-0.011), again reflecting the large difference in minor allele frequencies (**Supplementary Table 14**). The candidate genes were therefore prioritised on the basis of their own haplotype-trait associations, stem expression, and functional annotation rather than by LD with the peak SNP.

**Figure 7:**
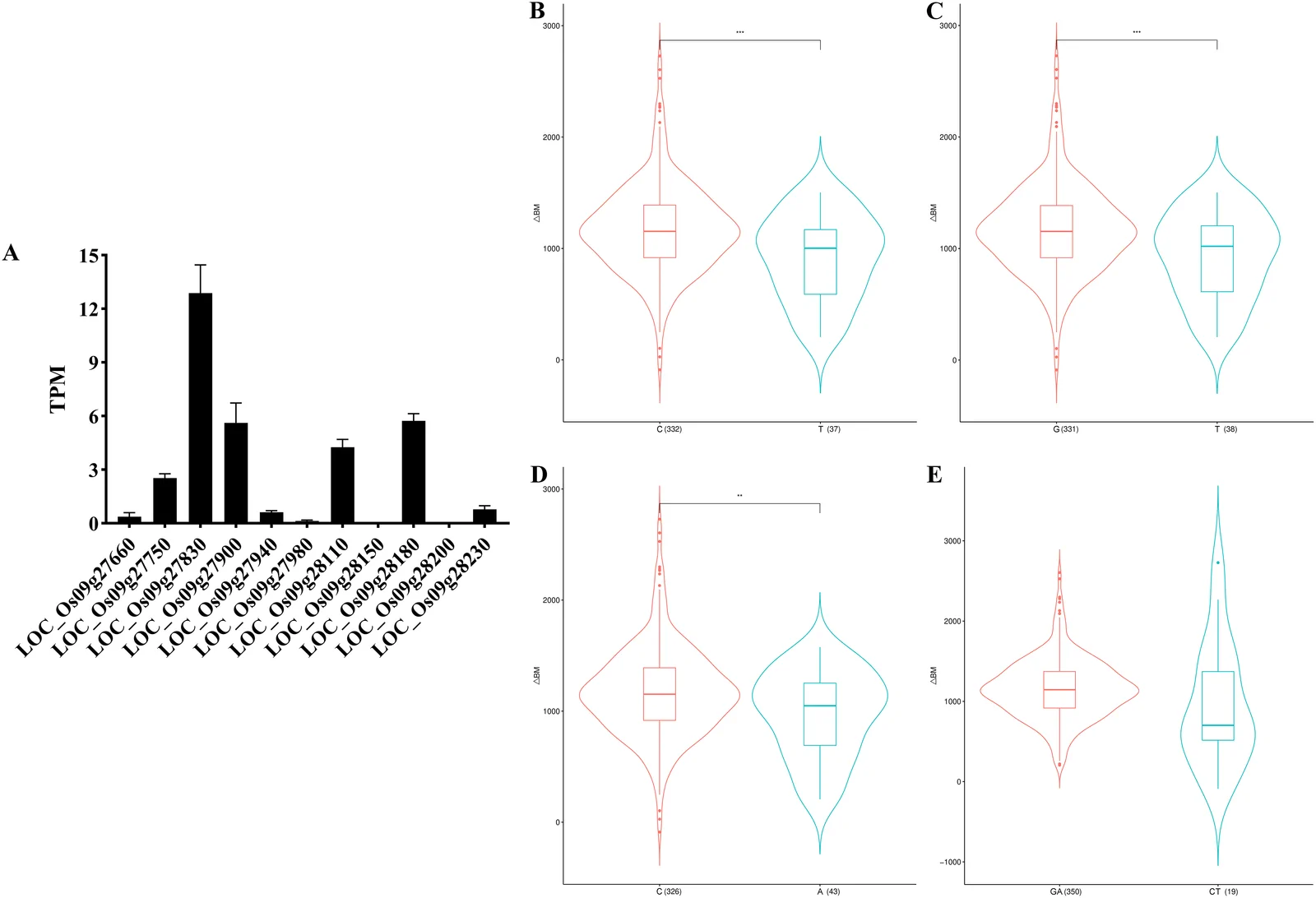
Stem expression levels of the 11 candidate genes based on the RNA-seq data of the Rice Genome Annotation Project **(A)** and haplotype analysis of the candidate genes *LOC_Os09g27830* **(B)**, *LOC_Os09g27900* **(C)**, *LOC_Os09g28110* **(D)** and *LOC_Os09g28180* **(E)** in *q△BM9* with BLUP. The x-axis labels indicate the SNP composition and haplotype counts. The y-axis shows the △BM values (g·cm). ^**^ *p* < 0.01, ^***^ *p* < 0.001 based on Student’s t-test.

## 4 Discussion

In this study, the eight selected lodging-related traits (PH, BR, FW, SL, SM, BS, LI, and △BM) of 370 *indica* rice accessions across three years and BLUP values were used to investigate the genetic architecture of culm lodging resistance. The observed significant phenotypic variation and near-normal frequency distribution of the studied traits indicated polygenic control, while high heritability values revealed substantial genetic potential for lodging resistance (**Table 1**; **Figure 3**). Among the eight selected lodging-related traits, we consider △BM as the most representative and comprehensive indicator of lodging resistance. Unlike LI, which is a ratio that can be influenced by both numerator and denominator effects, △BM directly quantifies the absolute mechanical capacity of the culm to withstand external forces (Luo et al., 2022b). It integrates information from both the bending moment at breaking (M) and the bending moment of the whole plant (BM), thereby capturing both intrinsic culm strength and the load imposed by the plant. Additionally, △BM showed high heritability and substantial phenotypic variation (**Table 1**), making it particularly suitable for genetic mapping. BR and BS are also excellent direct measures of mechanical strength, but they reflect only one aspect of lodging resistance without accounting for plant stature or biomass load. Therefore, for breeding applications aiming to improve overall lodging resistance, △BM provides the most integrated selection target.

To enable high-resolution GWAS, the panel has been deeply re-sequenced (average depth of approximately 11× per genome; NCBI BioProject PRJNA321462 and PRJNA331215), providing a marker density of 6.2 SNPs per kb and a mean SNP distance of 0.161 kb (**Supplementary Table 2**), which improved mapping resolution and imputation efficiency (Huang et al., 2010; Rashid et al., 2022a). In comparison, Du et al. (2022) employed 173 accessions at 5.48× depth and identified three candidate genes for flag leaf shape; Liu et al. (2023) mapped panicle-related genes using 5.48× coverage of 162 germplasms; and Wang et al. (2017) identified 72 QTL for grain appearance and milling quality using 22,488 SNPs with an average marker spacing of 16.6 kb. Therefore, this sequencing depth with dense markers was more than sufficient for GWAS, enabling robust mapping of candidate genes.

Furthermore, LD decay distance is another important factor in determining the GWAS resolution (Flint-Garcia et al., 2003; Myles et al., 2009; Hamblin et al., 2011; Lipka et al., 2015). Previous studies report that LD decay distance in *indica* rice populations ranged from 100 kb to 200 kb (McNally et al., 2009; Huang et al., 2011; Zhao et al., 2011; Lu et al., 2015; Yadav et al., 2015). In our study, the LD decay distance was approximately 92.95 kb (**Figure 1D**). This distance was shorter compared with previous reports, which was favored to locate the candidate genes. Generally, the reliability of GWAS results is determined by population structure (Yu et al., 2005; Uffelmann et al., 2021). Regardless of the population type, it was essential to estimate population structure prior to GWAS to avoid false positives and false negatives. In the current study, population structure analysis of the 370 *indica* rice accessions using PCA, kinship heatmap, and neighbor-joining tree revealed no strong or obvious population stratification; although a small group of accessions was distinguishable from the main population (**Figures 1A–C**), the overall structure was weak and was accounted for in all three GWAS models. As traditional single-locus model (MLM) has limitations such as neglecting small-effect loci, multi-locus models (FarmCPU and BLINK) could detect minor-effect loci along with improved computational efficiency (Zhang et al., 2018; Sachdeva et al., 2024). We applied all three models for GWAS of the eight selected lodging-related traits (**Figure 4**; **Supplementary Figures 1**-**3**). By incorporating the population structure detected by PCA and kinship into the association models and employing multiple models for association estimation, the occurrence of false positives and negatives was effectively controlled, which could be evidenced by the Q-Q plots (**Supplementary Figures 4**-**6**) (Kaler et al., 2020; Narawatthana et al., 2023).

Based on the above prerequisites that were highly suitable for GWAS, 10, 24, and 16 important QTLs were identified by the MLM, FarmCPU, and BLINK models, respectively (**Table 2**; **Figure 5**). In the *qPH1.1* region identified by the MLM, we found the cloned gene *LOC_Os01g66100* (*sd1*), which controls plant height (PH) by regulating the activity of gibberellin 20-oxidase, thereby affecting lodging resistance in rice (Maeda et al., 1997). Another cloned gene *OsBC1L4* encodes a COBRA-like protein affecting cellulose synthesis and stem weight in rice (Dai et al., 2011), which was located in the *qFW5* region identified by both the FarmCPU and BLINK models. Zhou et al. (2025) cloned the culm-strength gene *TC4*, which is located at approximately 30.18 Mb on chromosome 4, within the *qLI4.2* interval detected in the FarmCPU model. The *qSM3* region identified by the FarmCPU model was about 27 kb away from the pleiotropic gene *LOC_Os03g49880* (*SCM3, OsTB1*). This gene not only enhances lodging resistance by increasing the stem section modulus (SM), but also improves grain yield by increasing the spikelet number (Yano et al., 2015).

Besides above cloned genes, some previously fine-mapped QTLs were also identified. The *q△BM8* co-located with the previously reported *qSM8-1*, *qPR8-5*, and *qM8-6*, considered potential pleiotropic loci for SM, pushing resistance (PR), and bending moment at breaking (M) (Rashid et al., 2022b). The *qLI4.2* shared the same location with *qSWT4.3*, a major QTL for stem wall thickness (SWT) (Meng et al., 2021). The *qBR2* partially overlapped with a previous QTL, which was evaluated for M on chromosome 2 between markers RM1379 and RM3850 (Mulsanti et al., 2018). The *qPH12* fully overlapped with a previous QTL for PH in the region of 23.3-27.4 Mb on chromosome 12 (Kashiwagi and Ishimaru, 2004). The *qPH1* reported by Mamidi et al. (2026) lies within the *qPH1.1* interval detected in our study, providing further support for this region. Ookawa et al. (2016) detected QTLs for M and bending stress (BS) in the same genomic regions on chromosome 11. This region also harbored the pleiotropic QTLs, *qPH11*, *qLI11* and *q△BM11* (**Table 2**; **Figure 5**). The identification of these previously cloned genes and fine-mapped QTLs proved the accuracy and reliability of the GWAS results in the current research.

It should also be noted that *SCM3* (*LOC_Os03g49880*) (Yano et al., 2015) is located approximately 104 kb from the *qBS3* peak SNP, a distance comparable to the LD decay distance estimated in our panel. Although *SCM3* lies just outside the *qBS3* candidate interval and was therefore not included in our candidate-gene prioritization, the close proximity means that *qBS3* may not be fully independent of this well-characterized pleiotropic gene, whose known effect on SM could also influence BS. Fine-mapping and functional validation are required to clarify their relationship. Regarding the genetic position of *q△BM9*, its peak SNPs lie immediately adjacent to a pleiotropic QTL at approximately 16.30-16.91 Mb on chromosome 9 reported by Rashid et al. (2022b) for pushing resistance and M. Because △BM is a direct transform of M (△BM = 2M - BM), the *q△BM9* signal may correspond to the same locus as this previously reported QTL. Therefore, the novelty of *q△BM9* should be interpreted with caution, and fine-mapping is needed to determine whether the two signals are independent.

To further assess the robustness of the two stable QTLs, we repeated the multi-model GWAS for BS and △BM after applying a more stringent minor allele frequency threshold (MAF ≥ 0.10; 1,543,129 of 2,315,020 SNPs retained). Under this threshold, *q△BM9* remained genome-wide significant in the BLUP analysis in all three models and was also detected in individual years. In contrast, *qBS3* was not detected at MAF ≥ 0.10 in any model or year, because its peak SNP was removed by the MAF filter and no other SNP in the *qBS3* interval reached genome-wide significance under the threshold (**Supplementary Table 15**). These results confirm the robustness of *q△BM9* under a more stringent MAF threshold. For *qBS3*, whose peak SNP is too rare to be tested at MAF ≥ 0.10, its stability across the three models and across three years and BLUP at the original threshold remains the supporting evidence.

Regarding the three GWAS models employed, FarmCPU and BLINK demonstrated superior performance compared to the traditional MLM, as they detected more QTLs and identified QTLs with smaller effect sizes that were missed by MLM (**Table 2**; **Figure 5**). The multi-locus approach of FarmCPU and BLINK efficiently accounts for background polygenic effects and reduces false positives, as reflected in the well-controlled Q-Q plots (**Supplementary Figures 5**, **6**). However, the concurrent use of all three models remains the optimal strategy, as the QTLs (*qBS3* and *qΔBM9*) consistently identified across multiple models represent the most reliable targets for follow-up studies due to their robustness against model assumptions and population structure. This multi-model consensus approach is particularly valuable for complex traits like lodging resistance, which are controlled by both major and minor-effect loci.

Haplotype analysis and expression profiling revealed four candidate genes (*LOC_Os03g49570*, *LOC_Os03g49610*, *LOC_Os03g49710*, and *LOC_Os03g49730*) for *qBS3* and three candidate genes (*LOC_Os09g27830*, *LOC_Os09g27900*, and *LOC_Os09g28110*) for *q△BM9* (**Supplementary Table 7**). Among the *qBS3* candidates, *LOC_Os03g49610* (*Os3BGlu8*) emerged as the most promising, encoding a protein annotated as beta-glucosidase 8. Beta-glucosidase maintains the secondary structure of the cell wall by degrading cellulosic oligosaccharides and releasing lignin monomers from glycosides, potentially influencing the BS of stem (Kuntothom et al., 2009; Shim et al., 2022; Ketudat Cairns et al., 2023). Another most likely candidate gene for *q△BM9* was *LOC_Os09g28110*, encoding a hydroxyproline-rich glycoprotein family protein (HRGP). HRGPs form an insoluble three-dimensional network mainly through tyrosine covalent cross-linking of extensins and covalent linkages of arabinogalactan proteins (AGPs) with polysaccharides, directly affecting the mechanical strength and deformation resistance of the cell wall, which might influence the M of stem (Sommer-Knudsen et al., 1998; Deepak et al., 2010; Hijazi et al., 2014). It is worth noting that, in the publicly available RNA-seq data from the Rice Genome Annotation Project, neither *LOC_Os03g49610* nor *LOC_Os09g28110* exhibits the highest transcript abundance among all genes within their respective QTL intervals (**Figures 6A**, **7A**). However, our prioritisation was not based on expression abundance alone, but on an integrated assessment combining genetic association evidence, functional annotation, and detectable expression in stems. The significant phenotypic differences between haplotypes defined by non-synonymous SNPs provide more direct evidence of candidate gene relevance than expression abundance alone. Furthermore, a gene’s impact on complex traits is not always proportional to its mRNA level. *LOC_Os03g49610* encodes a beta-glucosidase, an enzyme that can process multiple substrate molecules per catalytic cycle, whereas *LOC_Os09g28110* encodes a hydroxyproline-rich glycoprotein, whose biological activity depends on precise spatial localisation and temporal deposition rather than bulk transcript abundance. Thus, even moderate expression levels can be sufficient for a key regulatory or structural role in cell wall architecture. Notably, the LD between the GWAS peak SNP and the non-synonymous variants of the candidate genes was low (*r*² = 0.003-0.011), reflecting the large difference in minor allele frequencies (**Supplementary Table 14**). Accordingly, the expression evidence concerns the candidate genes themselves and does not depend on the peak SNP. Based on these findings, we propose *LOC_Os03g49610* and *LOC_Os09g28110* as the most promising candidate genes for *qBS3* and *q△BM9*, respectively. This prioritization is supported by three lines of evidence: (1) they contain non-synonymous SNPs that define haplotypes with significantly different phenotypic values for BS and △BM, respectively; (2) they show relatively high expression levels in stem tissues according to publicly available RNA-seq data; (3) their annotated functions are plausibly linked to cell wall mechanical properties. However, because the non-synonymous SNPs across the candidate genes within each QTL are in strong LD (**Supplementary Table 13**), the individual contribution of each candidate gene cannot be fully separated by the current data; functional validations such as gene cloning, CRISPR/Cas9-mediated knockout, overexpression, and complementation (Wang et al., 2026) are necessary to confirm their biological roles in lodging resistance and elucidate the underlying molecular mechanisms.

Beyond dissecting the genetic architecture, the important QTLs and candidate genes identified in this study offer direct practical implications for rice breeding. First, the two stable QTLs, *qBS3* and *qΔBM9*, represent valuable targets for marker-assisted selection (MAS). The peak SNPs within these QTLs can be readily converted into kompetitive allele-specific PCR (KASP) markers, enabling breeders to introgress favorable alleles into elite rice varieties without compromising yield potential. Second, since both *qBS3* and *qΔBM9* affect mechanical parameters, pyramiding their favorable alleles through marker-assisted gene stacking may produce an additive or synergistic effect, further enhancing lodging resistance. Third, haplotype analysis has shown that specific haplotypes of *LOC_Os03g49610* and *LOC_Os09g28110* are significantly associated with superior mechanical traits. These diagnostic haplotypes can be used to screen parental lines and select elite progenies without the need for laborious phenotypic evaluations. Collectively, our findings not only provide mechanistic insights but also offer actionable genomic tools for the genetic improvement of lodging resistance in *indica* rice.

## 5 Conclusions

In the present study, we performed a multi-model GWAS on 370 *indica* rice accessions across three years and BLUP to dissect the genetic basis of lodging resistance. A total of 10, 24, and 16 important QTLs were identified by the MLM, FarmCPU, and BLINK models, respectively. Notably, two stable QTLs, *qBS3* and *q△BM9*, were consistently detected in all three models. Through haplotype analysis and expression profiling, we identified four candidate genes (*LOC_Os03g49570*, *LOC_Os03g49610*, *LOC_Os03g49710*, and *LOC_Os03g49730*) for *qBS3* and three candidate genes (*LOC_Os09g27830*, *LOC_Os09g27900*, and *LOC_Os09g28110*) for *q△BM9*. Among these, *LOC_Os03g49610* (encoding beta-glucosidase 8) and *LOC_Os09g28110* (encoding a hydroxyproline-rich glycoprotein) were proposed as promising candidate genes potentially affecting the bending stress and bending moment at breaking of the stem, respectively. Our findings provide valuable genetic resources for improving lodging resistance in rice breeding programs and offer insights into the molecular mechanisms underlying culm mechanical strength. Further functional validation of these candidate genes will be crucial for elucidating the regulatory networks of lodging resistance in *indica* rice. It should also be noted that *qBS3* and *q△BM9* are located in close proximity to previously characterized loci, whether these loci are independent of the earlier reports requires fine-mapping and functional validation.

## Data Availability

The datasets presented in this study can be found in online repositories. The names of the repository/repositories and accession number(s) can be found below: https://www.ncbi.nlm.nih.gov/, PRJNA321462 https://www.ncbi.nlm.nih.gov/, PRJNA331215.
