# Supplementary material for: Multi-model genome-wide association studies identify stable QTLs and candidate genes for lodging resistance in indica rice
Source: Front Plant Sci. 2026 Sep 18;17:1914726. doi: 10.3389/fpls.2026.1914726 (PMC13631065; doi:10.3389/fpls.2026.1914726)
Supplement: Supplementary file 1 [file SupplementaryFile1.docx]

Supplementary Material

# 1 Supplementary Figures and Tables

## 1.1 Supplementary Figures

**Supplementary Figure 1.** Manhattan plots of the GWAS results for the 8 selected lodging-related traits with MLM in 2023, 2024 and 2025. (A) PH; (B) SL; (C) BR; (D) SM; (E) BS; (F) △BM. The green horizontal line represents the genome-wide signiﬁcance threshold of Bonferroni adjusted *p* value. The dotted line represents the threshold of false discovery rate adjusted p value.

**Supplementary Figure 2.** Manhattan plots of the GWAS results for the 8 selected lodging-related traits with FarmCPU in 2023, 2024, 2025 and BLUP. (A) PH; (B) SL; (C) FW; (D) SM; (E) BS; (F) LI; (G) △BM. The green horizontal line represents the genome-wide signiﬁcance threshold of Bonferroni adjusted *p* value. The dotted line represents the threshold of false discovery rate adjusted *p* value.

**Supplementary Figure 3.** Manhattan plots of the GWAS results for the 8 selected lodging-related traits with BLINK in 2023, 2024, 2025 and BLUP. (A) PH; (B) SL; (C) FW; (D) BR; (E) BS; (F) LI; (G) △BM. The green horizontal line represents the genome-wide signiﬁcance threshold of Bonferroni adjusted *p* value. The dotted line represents the threshold of false discovery rate adjusted *p* value.

**Supplementary Figure 4.** Q-Q plots of the GWAS results for the 8 selected lodging-related traits with MLM in 2023, 2024, 2025 and BLUP. (A) PH; (B) SL; (C) BR; (D) SM; (E) BS; (F) △BM.

**Supplementary Figure 5.** Q-Q plots of the GWAS results for the 8 selected lodging-related traits with FarmCPU in 2023, 2024, 2025 and BLUP. (A) PH; (B) SL; (C) FW; (D) SM; (E) BS; (F) LI; (G) △BM.

**Supplementary Figure 6.** Q-Q plots of the GWAS results for the 8 selected lodging-related traits with BLINK in 2023, 2024, 2025 and BLUP. (A) PH; (B) SL; (C) FW; (D) BR; (E) BS; (F) LI; (G) △BM.

**Supplementary Figure 7.** Linkage disequilibrium (LD) heatmaps for the *qBS3* (A) and *q△BM9* (B) intervals. For each interval, the upper panel shows the association signals (−log_10_(*P*)) of the SNPs, with the peak SNP indicated; the lower panel shows pairwise *r*² values among SNPs.

## 1.2 Supplementary Tables

**Supplementary Table 1.** The information of 370 *indica* rice accessions.

| **Accession ID** | **Accession name** |
| --- | --- |
| A002 | Budda |
| A003 | Minghui63 |
| A004 | Chorofa |
| A005 | D11 |
| A006 | D15 |
| A007 | KR200 |
| A008 | 829 |
| A010 | RR 272-17-829 |
| A011 | K 479-2-3 |
| A012 | Zaoxian14 |
| A014 | IR65600-27-1-2-2 |
| A018 | Zhongyouzao81 |
| A019 | E ZI 96 |
| A020 | IRGA 370-38-1-1F-C4-2 |
| A022 | IR 2344-P1 PB-9-3-2B |
| A024 | ARROZVERMELHO |
| A028 | LALSAITA |
| A030 | DANAU LAUT TAWAR |
| A031 | IR 57920-AC-25-2-B |
| A032 | WAS 173-B-B-6-2-2 |
| A033 | ARAURE 1 |
| A035 | ICTA CRISPO 38 |
| A037 | IR77298-14-1-2 |
| A038 | Bala |
| A039 | IR55419-04 |
| A042 | UNNAMED |
| A044 | P59279 |
| A045 | Guang122 |
| A046 | IRAT144 |
| A047-req | 93072 |
| A048 | Maravilha |
| A050 | YZX1 |
| A051 | 42686 |
| A052 | TKM 9 |
| A054 | YA NONG ZAO 4 |
| A056 | BD007 |
| A057 | E 2024 |
| A058 | GLADIO |
| A060 | Y134 |
| A062 | Bg 300 |
| A064 | YZX2 |
| A066 | CALIFORNIA BELLE |
| A067 | WAS 206-B-B-2-2-1 |
| A068 | WAS 198-B-3-1-3 |
| A069 | IR 56 |
| A072 | IR8192-200-3-3-1-1 |
| A073 | BA SHI ZAO |
| A074 | Y075 |
| A075 | NERICA-L-27 |
| A076 | Molizhanxuan |
| A077 | Wanxian763 |
| A078 | CT 6510-24-1-2 |
| A079 | SONA (IET 1991) |
| A080 | Fengaizhan |
| A081 | Yuexiangzhan |
| A082 | Hnankar |
| A084 | OM1706 |
| A085 | IRBB7 |
| A086 | IR64-IL |
| A087 | IR68897B |
| A088 | PSBRC82 |
| A089 | NERICA-L-1 |
| A090 | Huhan15 |
| A093 | UPL RI-7 |
| A094 | B 6144-MR-6-0-0 |
| A095 | GIZA 178 |
| A096 | SAMBALA MALO |
| A098 | IRI 339 |
| A099 | IR 57514-PMI 5-B-1-2 |
| A101 | X21 |
| A102 | C70 |
| A103 | LX2007 |
| A104 | MAYBELLE |
| A105 | EX FOILAEIN (NAPUTO) |
| A107 | UPR 1201-1-20-1 |
| A109 | R644 |
| A111 | Carijo |
| A112 | IET1444 |
| A113 | Ganwanxian37 |
| A114 | Huangsizhan |
| A115 | IR 2003-P7-7-4-2 |
| A116 | SANHUANGZHAN NO 2 |
| A117 | IR 80310-12-B-1-3-B |
| A119 | Govind |
| A120 | NSIC RC9 (APO) |
| A121 | Hua565 |
| A122 | PSBRC80 |
| A124 | PT60 |
| A126 | BR11 |
| A127 | Huajingxian74 |
| A128 | IR66897B |
| A130 | Chhomromg |
| A131 | Palung 2 |
| A132 | Qb_604 |
| A134 | IR68 |
| A135 | SAGC-4 |
| A136 | Zhonghua1 |
| A137 | KCD1 |
| A140 | Shwe Thwe Yin Hyv |
| A141 | PSBRC88 |
| A142 | R106 |
| A144 | X23 |
| A146 | IRBB62 |
| A147 | ZH5 |
| A149 | NPT-100 |
| A153 | 2004 |
| A154 | At354 |
| A157 | Hua564 |
| A158 | E 2070 |
| A160 | Lai nok kha |
| A166 | LUO AI ZAO 3 |
| A167 | M 6034-1 |
| A169 | E ZI 32 |
| A173 | HUO QING |
| A177 | Chaoyang1B |
| A178 | Jinnante43B |
| A179 | Jiangnongzao1B |
| A181 | ZhuzhenB |
| A182 | Zaoxian240 |
| A185 | Gzhenshan97B |
| A186 | KANGRI |
| A198-req | N'YA NOFF |
| A199 | HONG YANG ZAO 3 |
| A203 | ZHAO DAN AI |
| A204 | Zhenfu8 |
| A208 | CN44-40-7 |
| A211 | KABERI |
| A212 | Sililanka1 |
| A213 | LIANG GUANG |
| A216 | W 398 |
| A217 | Nante |
| A218 | DA GU AI 7 |
| A219 | JIANG ER ZAO |
| A220 | PJ110 |
| A221 | GUANG XUAN LIU HAO |
| A222 | ZUIHOU |
| A223 | IR 77298-14-1-2-10 |
| A225 | Dianrui409B |
| A226 | 531 |
| A227 | FACAGRO 406 |
| A228 | QUN XUAN ZAO |
| A229 | IRAT 10 |
| A230 | Guangluai15-1 |
| A231 | Mamagu |
| A232 | ARC 11777 |
| A234 | E ZI 110 |
| A235 | Xiaohonggu |
| A236 | Dangyu5 |
| A238 | KUNJUKUNJU |
| A243-req | Momi |
| A245 | CUN GU NUO |
| A248 | QING TAI AI |
| A249 | PL 3165 |
| A250 | Heidu4 |
| A251 | Bawangbian1 |
| A253 | IR 19058-107-1 |
| A255 | RATNAGIRI 45-2 |
| A258 | B 737G-KN-23-1 |
| A259 | IR 73571-3B-11-3-K2 |
| A262 | Chengduai3 |
| A266 | Liusha1 |
| A268 | BuleidaA-75 |
| A270 | JIN HUA 258 |
| A271 | KA GIL |
| A272 | AR 133 |
| A273 | HSINCHU AI CHIO CHIENG |
| A275 | IR 77186-122-2-2-3 |
| A276 | Jiabala |
| A278 | JWR 221 |
| A280 | Gongju73 |
| A281 | Luke3 |
| A282 | BOUAKE 189 |
| A284 | NAN-ERH-AI 5 |
| A285 | SI CHAO 1 |
| A286 | TE SAN AI 2 |
| A288 | C 166-135 |
| A291 | TOS 9795 |
| A292 | K 24 |
| A293 | 13946 (GUANG-QIU 15) |
| A294 | RP 1570-44-1 |
| A296 | 3210 |
| A297 | INIAP 6 |
| A298 | CN1067 |
| A299 | IR 10120-7-2-1-4 |
| A303 | 71011 |
| A304 | TAISEN GLUTINOUS YU 1157 |
| A306 | Taizhongzailai1 |
| A309 | Menjiading2 |
| A310 | Gu154 |
| A311 | Kui630 |
| A312 | Taizhong65 (TaizhongHR539) |
| A314 | Padi Ladang Ase Polo Komek |
| A316 | LUBUK LINGGAU |
| A317 | Taizhongxianxuan2 |
| A319 | Meihuanuo |
| A320 | Muguanuo |
| A322 | ARC 7425 |
| A326 | SPR7284-57-5 |
| A327 | AUS 449 |
| A330-req | Xianghui91269 |
| A331 | R 762 |
| A332 | JHORA |
| A333 | SANTHI 206 |
| A335 | Aimakang |
| A342 | IR38（IR2070-423-2-5-6） |
| A344 | IR6 |
| A346 | Minghui86 |
| A347 | Kanghui63 |
| A348 | Luhui63 |
| A349 | Ce49 (Ce64-7-49) |
| A350 | Ce64 |
| A354 | Wanhui88 |
| A357 | Shengtai1 |
| A359 | Manghui |
| A360 | Zhenhui084 |
| A361 | R287 |
| A363 | Enhui69 |
| A365 | Ⅱ-32B |
| A367 | WuxiangB |
| A368 | V20B |
| A370 | MaxieB |
| A372 | 898B |
| A374 | EjinB |
| A375 | Ezao18 |
| A376 | Yue4B |
| A377 | Runzhu537 (Ezhong5) |
| A378 | Yuexiangzhan |
| A381 | Gaodanbaixiangdao |
| A382 | Jianzhen2 |
| A383 | BG304 |
| A384 | Bataixiangzhan |
| A387 | Miyang83 |
| A389 | Ewan13 |
| A390 | Wen229 |
| A391 | Zaoyou143 (Xiangzao143) |
| A392 | Yuanfengzao |
| A393 | Enhui58 |
| A394 | Guanghui380 |
| A395 | Guanghui880 |
| A396 | 158B |
| A397 | Jingchu814B |
| A399 | Gunong2923 |
| A400 | Guojisuo1 |
| A401 | Guooyou12 |
| A402 | #02428 |
| A403 | Zhaiyeqing8 |
| A404 | Forbiprotife |
| A405 | Ezao6 |
| A406 | Ezao11 |
| A408 | Zhenguiai |
| A409 | ShijinB |
| A412 | Digu |
| A418 | Aizaizhan |
| A419 | Guongchangai3784 |
| A421 | Jiangerai |
| A422 | Qingerai |
| A423 | Jiduilun |
| A424 | Kuoyedao |
| A425 | Yeqinglun |
| A426 | Lucai |
| A428 | Guangchangai4182 |
| A430 | Zhaoyangzao18 |
| A432 | Guangchang13 |
| A433 | Fengqingai |
| A434 | Qingnongai |
| A435 | Fengaizhan1 |
| A436 | Huanghuazhan |
| A437 | Huangxinzhan |
| A438 | 28zhan |
| A439 | Fenghuazhan |
| A440 | Qingliuai1 |
| A441 | Fengbaizhan |
| A442 | Changsizhan |
| A443 | Huasizhan |
| A450 | N22 |
| A451 | Aijiaonante |
| A452 | Guangluai4 |
| A453 | Xiangaizao10 |
| A456 | 88B |
| A464 | Guichao2 |
| A466 | Teqingxuanhui |
| A467 | Huangsiguizhan |
| A468 | Xiangwanxian3 |
| A469 | Jinyou1 |
| A470 | Chengnongshuijing |
| A472 | Sanbaili |
| A474 | Dongtingwanxian |
| A475 | Yangdao2 |
| A479 | Heidu4 |
| A481 | Xiangzaoxian7 |
| A482 | 80B |
| A483 | Baoxie123B |
| A486 | XiangaiB |
| A487 | Aituogu151 |
| A492 | Jiefangxian |
| A493 | Baikehualuo |
| A495 | Zhenshan97 |
| A503 | D62B |
| A508 | Xiang5 |
| A510 | Exiang1 |
| A511 | H198-yeza |
| A512 | Huangxiuzhan |
| A513 | Shuhui527 |
| A516 | Longhui11 |
| A517 | Nanhui511 |
| A519 | Jinhui275 |
| A520 | Luhui5240 |
| A521 | Minghui2088 |
| A522 | Shanhui287 |
| A523 | Shanhui8281 |
| A524 | R238 |
| A525 | R727 |
| A526 | 205R |
| A527 | 781R |
| A530 | IR1544 |
| A532 | Fu36-2 |
| A536 | Luhui17 |
| A538 | Yihui1577 |
| A539 | CDR22 |
| A540 | Shuhui202 |
| A541 | Shuhui881 |
| A542 | Mianhui725 |
| A543 | 299R |
| A544 | Shuhui162 |
| A545 | Chenghui727 |
| A547 | Xilong1R |
| A549 | Jinhui18 |
| A550 | Jinhui16 |
| A552 | Jinhui34 |
| A553 | Chuanhuihang907 |
| A555 | Jinhui35 |
| A557 | Wanhui66 |
| A558 | Fuhui802 |
| A563 | Chenghui881 |
| A565 | Kehui746 |
| A567 | Fuhui838 |
| A572 | Gui553 |
| A573 | Chenghui3203 |
| A577 | Huarun2 |
| A579 | Huazhan |
| A580 | Wusansimiao |
| A583 | E1573 |
| A585 | S112 |
| A595 | JAMBALI |
| A598 | IR 28 |
| A599 | - |
| A600 | C418 |
| A601 | KURULU WEE (WHITE) |
| A604 | SLAVA |
| A609 | DORELLA |
| A611 | Zhongchao123 |
| A614 | GRITNA |
| A616 | Gaozi |
| A619 | Zixiang3 |
| A622 | Zixiang2 |
| A623 | Hunanningxiangyuanzinuo |
| A624 | Hunanningxiangchangzinuo |
| A625 | Guangximomi |
| A629 | DYheidao |
| A631 | Jiangxishangraozixiangmi |
| A632 | Guangxishanglinhongxiangmi |
| A633 | Fujianyouxiheiguomi |
| A634 | Fujianyouxihongguomi |
| A637 | 15HP58 |
| A642 | R5431 |
| A643 | 99-14 |
| A644 | 900 |
| A645 | Feng9 |
| A647 | 9348 |
| A648 | YS21 |

**Supplementary Table 2.** Distributions of SNPs on chromosomes.

| **Chromosome** | **Size (bp)** | **SNP Number** | **SNP Distance (kb)** | **SNP Density (per kb)** |
| --- | --- | --- | --- | --- |
| 1 | 43,270,923 | 290,029 | 0.149 | 6.70 |
| 2 | 35,937,250 | 250,592 | 0.143 | 6.97 |
| 3 | 36,413,819 | 232,363 | 0.157 | 6.38 |
| 4 | 35,502,694 | 158,204 | 0.224 | 4.46 |
| 5 | 29,958,434 | 163,182 | 0.184 | 5.45 |
| 6 | 31,248,787 | 205,601 | 0.152 | 6.58 |
| 7 | 29,697,621 | 182,121 | 0.163 | 6.13 |
| 8 | 28,443,022 | 203,349 | 0.140 | 7.15 |
| 9 | 23,012,720 | 153,100 | 0.150 | 6.65 |
| 10 | 23,207,287 | 157,635 | 0.147 | 6.79 |
| 11 | 29,021,106 | 169,480 | 0.171 | 5.84 |
| 12 | 27,531,856 | 149,364 | 0.184 | 5.43 |
| Total | 373,245,519 | 2,315,020 | 0.161 | 6.20 |

**Supplementary Table 3.** SNPs signiﬁcantly associated with the 8 selected lodging-related traits identiﬁed by multi-model GWAS in 2023, 2024, 2025, and BLUP.

| **Model** | **Trait** | **SNP** | **Year** | **Chromosome** | **Position** | **MAF** | ***P* value** | **-log10(*P*)** | **FDR** |
| --- | --- | --- | --- | --- | --- | --- | --- | --- | --- |
| MLM | PH | chr01_38472986 | 2023 | 1 | 38,472,986 | 0.164865 | 8.21E-09 | 8.085584 | 0.003168 |
|  |  | chr01_38473000 | 2023 | 1 | 38,473,000 | 0.164865 | 8.21E-09 | 8.085584 | 0.003168 |
|  |  | chr01_38475032 | 2023 | 1 | 38,475,032 | 0.164865 | 8.21E-09 | 8.085584 | 0.003168 |
|  |  | chr01_38513991 | 2023 | 1 | 38,513,991 | 0.162162 | 1.4E-08 | 7.852408 | 0.004065 |
|  |  | chr01_38517394 | 2023 | 1 | 38,517,394 | 0.162162 | 1.4E-08 | 7.852408 | 0.004065 |
|  |  | chr12_23564042 | 2023 | 12 | 23,564,042 | 0.059459 | 7.31E-10 | 9.136216 | 0.001286 |
|  |  | chr12_23721929 | 2023 | 12 | 23,721,929 | 0.059459 | 1.67E-09 | 8.778063 | 0.001286 |
|  |  | chr12_23735950 | 2023 | 12 | 23,735,950 | 0.059459 | 1.67E-09 | 8.778063 | 0.001286 |
|  |  | chr01_39171215 | 2024 | 1 | 39,171,215 | 0.308108 | 2.8E-09 | 8.552808 | 0.005644 |
|  |  | chr12_23564042 | 2025 | 12 | 23,564,042 | 0.059459 | 8.18E-09 | 8.087219 | 0.00163 |
|  |  | chr01_38472986 | BLUP | 1 | 38,472,986 | 0.164865 | 8.65E-09 | 8.063031 | 0.003025 |
|  |  | chr01_38473000 | BLUP | 1 | 38,473,000 | 0.164865 | 8.65E-09 | 8.063031 | 0.003025 |
|  |  | chr01_38475032 | BLUP | 1 | 38,475,032 | 0.164865 | 8.65E-09 | 8.063031 | 0.003025 |
|  |  | chr01_38513991 | BLUP | 1 | 38,513,991 | 0.162162 | 1.01E-08 | 7.996475 | 0.003025 |
|  |  | chr01_38517394 | BLUP | 1 | 38,517,394 | 0.162162 | 1.01E-08 | 7.996475 | 0.003025 |
|  |  | chr01_39171215 | BLUP | 1 | 39,171,215 | 0.308108 | 2.13E-08 | 7.672068 | 0.003025 |
|  |  | chr01_39518252 | BLUP | 1 | 39,518,252 | 0.078378 | 1.86E-08 | 7.731361 | 0.003025 |
|  |  | chr12_23564042 | BLUP | 12 | 23,564,042 | 0.059459 | 6.82E-09 | 8.166327 | 0.003025 |
|  | SL | chr12_23165720 | 2025 | 12 | 23,165,720 | 0.070270 | 2.18E-11 | 10.66245 | 5.04E-05 |
|  |  | chr12_23176767 | 2025 | 12 | 23,176,767 | 0.059459 | 1.52E-08 | 7.818401 | 0.005861 |
|  |  | chr12_23181706 | 2025 | 12 | 23,181,706 | 0.067568 | 1.24E-08 | 7.906701 | 0.00574 |
|  |  | chr12_23564042 | 2025 | 12 | 23,564,042 | 0.059459 | 8.2E-09 | 8.086402 | 0.004743 |
|  |  | chr12_23721929 | 2025 | 12 | 23,721,929 | 0.059459 | 1.28E-09 | 8.891415 | 0.000991 |
|  |  | chr12_23735950 | 2025 | 12 | 23,735,950 | 0.059459 | 1.28E-09 | 8.891415 | 0.000991 |
|  |  | chr12_23165720 | BLUP | 12 | 23,165,720 | 0.070270 | 3.55E-09 | 8.450205 | 0.001651 |
|  |  | chr12_23564042 | BLUP | 12 | 23,564,042 | 0.059459 | 1.73E-08 | 7.762007 | 0.001651 |
|  |  | chr12_23721929 | BLUP | 12 | 23,721,929 | 0.059459 | 8.19E-09 | 8.086628 | 0.001651 |
|  |  | chr12_23735950 | BLUP | 12 | 23,735,950 | 0.059459 | 8.19E-09 | 8.086628 | 0.001651 |
|  | BR | chr09_5382770 | 2024 | 9 | 5,382,770 | 0.070270 | 1.44E-08 | 7.840136 | 0.016726 |
|  |  | chr09_5382834 | 2024 | 9 | 5,382,834 | 0.070270 | 1.44E-08 | 7.840136 | 0.016726 |
|  | SM | chr02_26392548 | 2024 | 2 | 26,392,548 | 0.113514 | 2.14E-08 | 7.668593 | 0.049655 |
|  | BS | chr03_28324322 | BLUP | 3 | 28,324,322 | 0.051351 | 6.9E-09 | 8.160915 | 0.009665 |
|  |  | chr07_17628452 | BLUP | 7 | 17,628,452 | 0.051351 | 8.35E-09 | 8.078311 | 0.009665 |
|  |  | chr07_17634270 | BLUP | 7 | 17,634,270 | 0.070270 | 1.97E-08 | 7.704569 | 0.015236 |
|  | △BM | chr09_17059241 | 2024 | 9 | 17,059,241 | 0.078378 | 1.72E-08 | 7.765494 | 0.002572 |
|  |  | chr09_16911051 | BLUP | 9 | 16,911,051 | 0.075676 | 4.85E-09 | 8.31384 | 0.000836 |
|  |  | chr09_16917568 | BLUP | 9 | 16,917,568 | 0.113514 | 1.04E-08 | 7.982329 | 0.000836 |
|  |  | chr09_16922008 | BLUP | 9 | 16,922,008 | 0.075676 | 4.85E-09 | 8.31384 | 0.000836 |
|  |  | chr09_16961271 | BLUP | 9 | 16,961,271 | 0.075676 | 4.85E-09 | 8.31384 | 0.000836 |
|  |  | chr09_16962092 | BLUP | 9 | 16,962,092 | 0.113514 | 1.04E-08 | 7.982329 | 0.000836 |
|  |  | chr09_16962096 | BLUP | 9 | 16,962,096 | 0.113514 | 1.04E-08 | 7.982329 | 0.000836 |
|  |  | chr09_16965423 | BLUP | 9 | 16,965,423 | 0.075676 | 4.85E-09 | 8.31384 | 0.000836 |
|  |  | chr09_17012752 | BLUP | 9 | 17,012,752 | 0.075676 | 4.85E-09 | 8.31384 | 0.000836 |
|  |  | chr09_17059241 | BLUP | 9 | 17,059,241 | 0.078378 | 2.91E-09 | 8.536058 | 0.000836 |
|  |  | chr09_17089341 | BLUP | 9 | 17,089,341 | 0.116216 | 1.6E-08 | 7.795043 | 0.000836 |
|  |  | chr09_17089966 | BLUP | 9 | 17,089,966 | 0.113514 | 1.04E-08 | 7.982329 | 0.000836 |
|  |  | chr09_17089987 | BLUP | 9 | 17,089,987 | 0.113514 | 1.04E-08 | 7.982329 | 0.000836 |
| FarmCPU | PH | chr04_4776418 | 2023 | 4 | 4,776,418 | 0.191892 | 1.97E-08 | 7.706096 | 0.015182 |
|  |  | chr06_27524080 | 2023 | 6 | 27,524,080 | 0.081081 | 1.14E-08 | 7.94373 | 0.013176 |
|  |  | chr09_19256151 | 2023 | 9 | 19,256,151 | 0.078378 | 7.57E-12 | 11.12063 | 1.75E-05 |
|  |  | chr01_39171215 | 2024 | 1 | 39,171,215 | 0.308108 | 2.53E-13 | 12.59637 | 5.86E-07 |
|  |  | chr03_20115880 | 2024 | 3 | 20,115,880 | 0.113514 | 1.81E-09 | 8.7432 | 0.000836 |
|  |  | chr04_4776232 | 2024 | 4 | 4,776,232 | 0.332432 | 9.33E-11 | 10.03017 | 7.2E-05 |
|  |  | chr07_10251892 | 2024 | 7 | 10,251,892 | 0.140541 | 1.15E-12 | 11.94001 | 1.33E-06 |
|  |  | chr08_18180288 | 2024 | 8 | 18,180,288 | 0.067568 | 1.02E-09 | 8.99229 | 0.000589 |
|  |  | chr02_2827636 | 2025 | 2 | 2,827,636 | 0.251351 | 1.63E-08 | 7.788311 | 0.037692 |
|  |  | chr01_39171215 | BLUP | 1 | 39,171,215 | 0.308108 | 2.14E-11 | 10.66862 | 4.97E-05 |
|  |  | chr03_23007861 | BLUP | 3 | 23,007,861 | 0.118919 | 1.11E-08 | 7.953729 | 0.012876 |
|  | SL | chr03_20115880 | 2023 | 3 | 20,115,880 | 0.113514 | 2.74E-09 | 8.562095 | 0.006345 |
|  |  | chr03_12250984 | 2025 | 3 | 12,250,984 | 0.227027 | 9.76E-09 | 8.010716 | 0.012122 |
|  |  | chr03_35024514 | 2025 | 3 | 35,024,514 | 0.054054 | 1.05E-08 | 7.979949 | 0.012122 |
|  | FW | chr02_29692818 | 2024 | 2 | 29,692,818 | 0.154054 | 2.12E-11 | 10.67405 | 2.7E-05 |
|  |  | chr06_30161651 | 2024 | 6 | 30,161,651 | 0.054054 | 2.33E-11 | 10.63194 | 2.7E-05 |
|  |  | chr02_30359901 | 2025 | 2 | 30,359,901 | 0.429730 | 1.87E-15 | 14.72799 | 4.33E-09 |
|  |  | chr03_5527524 | 2025 | 3 | 5,527,524 | 0.059459 | 1.77E-09 | 8.750982 | 0.000685 |
|  |  | chr05_18677505 | 2025 | 5 | 18,677,505 | 0.067568 | 1.33E-14 | 13.87774 | 1.53E-08 |
|  |  | chr08_3957026 | 2025 | 8 | 3,957,026 | 0.072973 | 1.16E-10 | 9.93391 | 6.74E-05 |
|  |  | chr09_20007029 | 2025 | 9 | 20,007,029 | 0.289189 | 4.94E-11 | 10.30619 | 3.81E-05 |
|  |  | chr11_21642276 | 2025 | 11 | 21,642,276 | 0.070270 | 1.99E-10 | 9.701536 | 9.21E-05 |
|  |  | chr02_20114332 | BLUP | 2 | 20,114,332 | 0.270270 | 6.97E-09 | 8.156927 | 0.008065 |
|  |  | chr05_18677505 | BLUP | 5 | 18,677,505 | 0.067568 | 1.86E-10 | 9.73103 | 0.00043 |
|  | SM | chr04_3936283 | 2023 | 4 | 3,936,283 | 0.132432 | 1.43E-09 | 8.844069 | 0.003315 |
|  |  | chr09_17225506 | 2023 | 9 | 17,225,506 | 0.124324 | 1.41E-08 | 7.849795 | 0.016358 |
|  |  | chr06_30161651 | 2024 | 6 | 30,161,651 | 0.054054 | 1.12E-09 | 8.951323 | 0.00259 |
|  |  | chr01_33328752 | BLUP | 1 | 33,328,752 | 0.143243 | 1.38E-08 | 7.861233 | 0.015933 |
|  |  | chr03_28550819 | BLUP | 3 | 28,550,819 | 0.054054 | 6.07E-10 | 9.216981 | 0.001405 |
|  | BS | chr03_28324322 | 2023 | 3 | 28,324,322 | 0.051351 | 1.06E-08 | 7.976527 | 0.012218 |
|  |  | chr10_2418048 | 2023 | 10 | 2,418,048 | 0.078378 | 2.96E-10 | 9.528033 | 0.000686 |
|  |  | chr10_10028635 | 2024 | 10 | 10,028,635 | 0.059459 | 5.95E-10 | 9.225537 | 0.001377 |
|  |  | chr11_26664590 | 2024 | 11 | 26,664,590 | 0.100000 | 1.78E-08 | 7.750223 | 0.020573 |
|  |  | chr03_28324322 | BLUP | 3 | 28,324,322 | 0.051351 | 2.14E-11 | 10.66886 | 4.96E-05 |
|  |  | chr07_26131378 | BLUP | 7 | 26,131,378 | 0.302703 | 6.6E-09 | 8.180703 | 0.007635 |
|  | LI | chr04_5932029 | 2023 | 4 | 5,932,029 | 0.289189 | 1.14E-09 | 8.941577 | 0.002648 |
|  |  | chr07_20873875 | 2023 | 7 | 20,873,875 | 0.159459 | 6.24E-09 | 8.205064 | 0.007219 |
|  |  | chr03_18553982 | 2024 | 3 | 18,553,982 | 0.094595 | 1.03E-10 | 9.988863 | 0.000119 |
|  |  | chr07_9154327 | 2024 | 7 | 9,154,327 | 0.251351 | 4.17E-11 | 10.37972 | 9.66E-05 |
|  |  | chr08_858309 | 2024 | 8 | 858,309 | 0.086486 | 7.64E-09 | 8.117005 | 0.005894 |
|  |  | chr04_23605597 | BLUP | 4 | 23,605,597 | 0.054054 | 1.52E-09 | 8.81819 | 0.001835 |
|  |  | chr04_30195183 | BLUP | 4 | 30,195,183 | 0.213514 | 1.58E-09 | 8.799972 | 0.001835 |
|  |  | chr10_17535744 | BLUP | 10 | 17,535,744 | 0.078378 | 6.87E-09 | 8.163042 | 0.005301 |
|  | △BM | chr09_16911051 | 2023 | 9 | 16,911,051 | 0.075676 | 9.83E-15 | 14.00746 | 2.28E-08 |
|  |  | chr10_774638 | 2023 | 10 | 774,638 | 0.056757 | 3.31E-09 | 8.480126 | 0.003832 |
|  |  | chr09_16911051 | 2024 | 9 | 16,911,051 | 0.075676 | 6.59E-13 | 12.18142 | 1.52E-06 |
|  |  | chr09_20967791 | 2024 | 9 | 20,967,791 | 0.100000 | 1.18E-08 | 7.929673 | 0.01361 |
|  |  | chr07_28381685 | 2025 | 7 | 28,381,685 | 0.075676 | 1.16E-09 | 8.936271 | 0.001197 |
|  |  | chr09_17692287 | 2025 | 9 | 17,692,287 | 0.116216 | 8.72E-09 | 8.059345 | 0.005048 |
|  |  | chr10_1538951 | 2025 | 10 | 1,538,951 | 0.335135 | 1.55E-09 | 8.809178 | 0.001197 |
|  |  | chr11_3074474 | 2025 | 11 | 3,074,474 | 0.102703 | 7.74E-11 | 10.11142 | 0.000179 |
|  |  | chr08_19418487 | BLUP | 8 | 19,418,487 | 0.097297 | 6.01E-09 | 8.221414 | 0.004635 |
|  |  | chr09_16911051 | BLUP | 9 | 16,911,051 | 0.075676 | 3.27E-18 | 17.48589 | 7.56E-12 |
|  |  | chr11_3074474 | BLUP | 11 | 3,074,474 | 0.102703 | 3.88E-15 | 14.41165 | 4.49E-09 |
| Blink | PH | chr07_12843936 | 2024 | 7 | 12,843,936 | 0.137838 | 1.35E-09 | 8.869296 | 0.001564 |
|  |  | chr11_3091039 | 2024 | 11 | 3,091,039 | 0.102703 | 3.38E-10 | 9.470534 | 0.000783 |
|  | SL | chr01_40776045 | 2025 | 1 | 40,776,045 | 0.108108 | 2.42E-09 | 8.615367 | 0.005613 |
|  |  | chr10_13552733 | BLUP | 10 | 13,552,733 | 0.237838 | 5.44E-09 | 8.26454 | 0.01259 |
|  | FW | chr09_11634593 | 2024 | 9 | 11,634,593 | 0.281081 | 5.7E-09 | 8.244335 | 0.013189 |
|  |  | chr02_30359901 | 2025 | 2 | 30,359,901 | 0.429730 | 5.01E-11 | 10.30055 | 0.000116 |
|  |  | chr05_18677505 | 2025 | 5 | 18,677,505 | 0.067568 | 5.22E-09 | 8.281926 | 0.004032 |
|  |  | chr08_3957026 | 2025 | 8 | 3,957,026 | 0.072973 | 1.2E-08 | 7.922194 | 0.006923 |
|  |  | chr09_20007081 | 2025 | 9 | 20,007,081 | 0.291892 | 2.38E-09 | 8.623965 | 0.002751 |
|  |  | chr02_30412829 | BLUP | 2 | 30,412,829 | 0.440541 | 2.08E-08 | 7.681485 | 0.016068 |
|  |  | chr09_11634593 | BLUP | 9 | 11,634,593 | 0.281081 | 1.64E-08 | 7.784367 | 0.016068 |
|  |  | chr11_20333086 | BLUP | 11 | 20,333,086 | 0.135135 | 1.96E-10 | 9.708019 | 0.000453 |
|  | BR | chr02_20048231 | 2024 | 2 | 20,048,231 | 0.400000 | 5.59E-10 | 9.252313 | 0.001295 |
|  |  | chr09_5382770 | 2024 | 9 | 5,382,770 | 0.070270 | 7.34E-09 | 8.134258 | 0.005665 |
|  |  | chr09_5382834 | 2024 | 9 | 5,382,834 | 0.070270 | 7.34E-09 | 8.134258 | 0.005665 |
|  |  | chr02_20044273 | BLUP | 2 | 20,044,273 | 0.397297 | 3.06E-10 | 9.513976 | 0.000709 |
|  |  | chr09_5382770 | BLUP | 9 | 5,382,770 | 0.070270 | 1.67E-08 | 7.776793 | 0.008785 |
|  |  | chr09_5382834 | BLUP | 9 | 5,382,834 | 0.070270 | 1.67E-08 | 7.776793 | 0.008785 |
|  | BS | chr08_19659717 | 2024 | 8 | 19,659,717 | 0.059459 | 7.47E-10 | 9.126557 | 0.00173 |
|  |  | chr03_28324322 | 2025 | 3 | 28,324,322 | 0.051351 | 1.1E-08 | 7.958707 | 0.025459 |
|  | LI | chr11_3074474 | 2025 | 11 | 3,074,474 | 0.102703 | 6.88E-11 | 10.16273 | 0.000159 |
|  |  | chr11_3074474 | BLUP | 11 | 3,074,474 | 0.102703 | 3.17E-09 | 8.499301 | 0.007333 |
|  |  | chr12_24022190 | BLUP | 12 | 24,022,190 | 0.143243 | 1.34E-08 | 7.873349 | 0.015494 |
|  | △BM | chr01_31005781 | 2023 | 1 | 31,005,781 | 0.059459 | 3.54E-11 | 10.45116 | 4.1E-05 |
|  |  | chr09_16911051 | 2023 | 9 | 16,911,051 | 0.075676 | 4.45E-15 | 14.35165 | 1.03E-08 |
|  |  | chr12_26108479 | 2023 | 12 | 26,108,479 | 0.210811 | 6.88E-09 | 8.162708 | 0.005305 |
|  |  | chr09_16911051 | 2024 | 9 | 16,911,051 | 0.075676 | 7.14E-13 | 12.14632 | 1.65E-06 |
|  |  | chr07_28381685 | 2025 | 7 | 28,381,685 | 0.075676 | 1.22E-09 | 8.912835 | 0.00283 |
|  |  | chr09_16911051 | BLUP | 9 | 16,911,051 | 0.075676 | 9.96E-17 | 16.00192 | 2.3E-10 |
|  |  | chr11_3074474 | BLUP | 11 | 3,074,474 | 0.102703 | 6.55E-11 | 10.18384 | 7.58E-05 |

MAF, minor allele frequency; FDR, false discovery rate.

**Supplementary Table 4.** Effect sizes and variance explained for the stable QTLs *qBS3* and *q△BM9* in 2023, 2024, 2025, and BLUP.

| **QTL** | **Model** | **Year** | **Peak_SNP** | **MAF** | ***P* value** | **FDR** | **Effect** | **△R² (%)** | **Notes** |
| --- | --- | --- | --- | --- | --- | --- | --- | --- | --- |
| *qBS3* | MLM | BLUP | chr03_28324322 | 0.051 | 6.90E-09 | 9.67E-03 | 87.13 | 6.95 |  |
|  | FarmCPU | 2023 |  |  | 1.06E-08 | 1.22E-02 | 93.08 | 5.49 | △R² from MLM-2023 same SNP |
|  |  | BLUP |  |  | 2.14E-11 | 4.96E-05 | 65.11 | 6.95 | △R² from MLM-BLUP same SNP |
|  | BLINK | 2025 |  |  | 1.10E-08 | 2.55E-02 | 87.13 | 6.95 | effect/△R² from MLM-BLUP same SNP |
| *q△BM9* | MLM | 2024 | chr09_17059241 | 0.078 | 1.72E-08 | 2.57E-03 | 743.85 | 7.86 |  |
|  |  | BLUP |  |  | 2.91E-09 | 8.36E-04 | 474.24 | 7.51 |  |
|  | FarmCPU | 2023 | chr09_16911051 | 0.076 | 9.83E-15 | 2.28E-08 | 653.51 | 6.37 | △R² from MLM-2023 same SNP |
|  |  | 2024 |  |  | 6.59E-13 | 1.52E-06 | 657.36 | 7.68 | △R² from MLM-2024 same SNP |
|  |  | BLUP |  |  | 3.27E-18 | 7.56E-12 | 440.48 | 7.29 | △R² from MLM-BLUP same SNP |
|  | BLINK | 2023 |  |  | 4.45E-15 | 1.03E-08 | 757.32 | 6.37 | effect/△R² from MLM-2023 same SNP |
|  |  | 2024 |  |  | 7.14E-13 | 1.65E-06 | 768.24 | 7.68 | effect/△R² from MLM-2024 same SNP |
|  |  | BLUP |  |  | 9.96E-17 | 2.30E-10 | 495.86 | 7.29 | effect/△R² from MLM-BLUP same SNP |

△R² (%) is the increase in model R² attributable to the peak SNP. For FarmCPU and BLINK rows, the △R² and, where BLINK does not provide it, the effect are estimated from the MLM for the same peak SNP (see notes).

**Supplementary Table 5.** Functional annotation of candidate genes in *qBS3*.

| **Number** | **Gene ID** | **Position (bp)** | **Functional annotation** |
| --- | --- | --- | --- |
| 1 | *LOC_Os03g49570* | 28229288 - 28234093 | domain of unknown function, DUF250 domain containing protein, expressed |
| 2 | *LOC_Os03g49580* | 28236193 - 28239377 | eukaryotic peptide chain release factor subunit 1-1, putative, expressed |
| 3 | *LOC_Os03g49600* | 28245347 - 28240285 | Os3bglu7 - beta-glucosidase, exo-beta-glucanase, expressed |
| 4 | *LOC_Os03g49610* | 28254047 - 28248731 | Os3bglu8 - beta-glucosidase, exo-beta-glucanase, high similarity to Os3bglu7, expressed |
| 5 | *LOC_Os03g49620* | 28258987 - 28254433 | BRASSINOSTEROID INSENSITIVE 1-associated receptor kinase 1 precursor, putative, expressed |
| 6 | *LOC_Os03g49640* | 28275709 - 28269518 | STE_MEKK_ste11_MAP3K.14 - STE kinases include homologs to sterile 7, sterile 11 and sterile 20 from yeast, expressed |
| 7 | *LOC_Os03g49700* | 28297142 - 28300290 | SNARE associated Golgi protein, putative, expressed |
| 8 | *LOC_Os03g49710* | 28301288 - 28303246 | ribosomal protein S13p/S18e, putative, expressed |
| 9 | *LOC_Os03g49720* | 28304985 - 28307930 | PAP fibrillin family domain containing protein, expressed |
| 10 | *LOC_Os03g49730* | 28312269 - 28308340 | zinc finger protein, putative, expressed |
| 11 | *LOC_Os03g49750* | 28314271 - 28319959 | protein kinase family protein, putative, expressed |
| 12 | *LOC_Os03g49770* | 28329951 - 28339179 | oxysterol-binding protein, putative, expressed |
| 13 | *LOC_Os03g49790* | 28345433 - 28346719 | pattern formation protein EMB30, putative, expressed |
| 14 | *LOC_Os03g49800* | 28362611 - 28358095 | phosphatidylinositol-4-phosphate 5-kinase, putative, expressed |

**Supplementary Table 6.** Functional annotation of candidate genes in *q△BM9*.

| **Number** | **Gene ID** | **Position (bp)** | **Functional annotation** |
| --- | --- | --- | --- |
| 1 | *LOC_Os09g27650* | 16822234 - 16825686 | ZOS9-14 - C2H2 zinc finger protein, expressed |
| 2 | *LOC_Os09g27660* | 16829409 - 16837307 | OsFBO21 - F-box and other domain containing protein, expressed |
| 3 | *LOC_Os09g27700* | 16861064 - 16855316 | microtubule associated protein, putative, expressed |
| 4 | *LOC_Os09g27710* | 16867460 - 16868178 | pollen allergen Cyn d 23, putative, expressed |
| 5 | *LOC_Os09g27730* | 16875521 - 16876343 | HVA22, putative, expressed |
| 6 | *LOC_Os09g27750* | 16887617 - 16886019 | 1-aminocyclopropane-1-carboxylate oxidase 1, putative, expressed |
| 7 | *LOC_Os09g27820* | 16925457 - 16927082 | 1-aminocyclopropane-1-carboxylate oxidase protein, putative, expressed |
| 8 | *LOC_Os09g27830* | 16930987 - 16935970 | OsPDIL2-3 protein disulfide isomerase PDIL2-3, expressed |
| 9 | *LOC_Os09g27850* | 16941833 - 16943438 | transcription regulator, putative, expressed |
| 10 | *LOC_Os09g27890* | 16958544 - 16962181 | lysM domain-containing GPI-anchored protein precursor, putative, expressed |
| 11 | *LOC_Os09g27900* | 16963593 - 16965490 | plastidic glucose transporter 1, putative, expressed |
| 12 | *LOC_Os09g27910* | 16970180 - 16971732 | aspartic proteinase nepenthesin precursor, putative, expressed |
| 13 | *LOC_Os09g27930* | 16973690 - 16975737 | ubiquitin fusion protein, putative, expressed |
| 14 | *LOC_Os09g27940* | 16977482 - 16980623 | aspartic proteinase nepenthesin-1 precursor, putative, expressed |
| 15 | LOC_Os09g27950 | 16983978 - 16979991 | galactosyltransferase, putative, expressed |
| 16 | LOC_Os09g27960 | 16990467 - 16987939 | transmembrane protein 50A, putative, expressed |
| 17 | LOC_Os09g27980 | 16996540 - 16999938 | emp24/gp25L/p24 family protein, putative, expressed |
| 18 | LOC_Os09g27990 | 17001374 - 16999259 | annexin, putative, expressed |
| 19 | LOC_Os09g28000 | 17005694 - 17003030 | OsSigP6 - Putative Type I Signal Peptidase homologue%3B employs a putative Ser/Lys catalytic dyad, expressed |
| 20 | LOC_Os09g28019 | 17014365 - 17008658 | nucleoporin p58/p45, putative, expressed |
| 21 | LOC_Os09g28040 | 17019853 - 17015450 | OsPOP18 - Putative Prolyl Oligopeptidase homologue, expressed |
| 22 | LOC_Os09g28050 | 17024571 - 17028551 | asparate aminotransferase, putative, expressed |
| 23 | LOC_Os09g28060 | 17035510 - 17033402 | MORN repeat domain containing protein, expressed |
| 24 | LOC_Os09g28100 | 17059795 - 17062359 | OsRhmbd15 - Putative Rhomboid homologue, expressed |
| 25 | LOC_Os09g28110 | 17067608 - 17062565 | hydroxyproline-rich glycoprotein family protein, putative, expressed |
| 26 | LOC_Os09g28120 | 17071783 - 17074131 | OsFBX330 - F-box domain containing protein, expressed |
| 27 | LOC_Os09g28130 | 17075709 - 17075190 | carbonic anhydrase family protein, putative, expressed |
| 28 | LOC_Os09g28150 | 17079644 - 17077665 | bifunctional monodehydroascorbate reductase and carbonic anhydrase/nectarin-3 precursor, putative, expressed |
| 29 | LOC_Os09g28160 | 17082095 - 17083568 | phosphate carrier protein, mitochondrial precursor, putative, expressed |
| 30 | LOC_Os09g28180 | 17096006 - 17091951 | D-mannose binding lectin family protein, expressed |
| 31 | LOC_Os09g28200 | 17111077 - 17109289 | HSF-type DNA-binding domain containing protein, expressed |
| 32 | LOC_Os09g28210 | 17132211 - 17133476 | bHelix-loop-helix transcription factor, putative, expressed |
| 33 | LOC_Os09g28220 | 17141366 - 17143325 | NFKB, putative, expressed |
| 34 | LOC_Os09g28230 | 17147124 - 17145734 | gibberellin receptor GID1L2, putative, expressed |
| 35 | LOC_Os09g28280 | 17172815 - 17171657 | gibberellin receptor GID1L2, putative, expressed |
| 36 | LOC_Os09g28300 | 17182867 - 17188378 | remorin C-terminal domain containing protein, putative, expressed |

**Supplementary Table 7.** Candidate genes harboring non-synonymous mutations in *qBS3* and *q△BM9.*

| **QTL** | **Chromosome** | **Genomic interval** | **Gene ID** | **Functional annotation** |
| --- | --- | --- | --- | --- |
| *qBS3* | 3 | 28,231,368-28,417,276 | *LOC_Os03g49570* | domain of unknown function, DUF250 domain containing protein, expressed |
|  |  |  | *LOC_Os03g49610* | Os3bglu8 - beta-glucosidase, exo-beta-glucanase, high similarity to Os3bglu7, expressed |
|  |  |  | *LOC_Os03g49710* | ribosomal protein S13p/S18e, putative, expressed |
|  |  |  | *LOC_Os03g49730* | zinc finger protein, putative, expressed |
| *q△BM9* | 9 | 16,818,097-17,182,941 | *LOC_Os09g27660* | OsFBO21 - F-box and other domain containing protein, expressed |
|  |  |  | *LOC_Os09g27750* | 1-aminocyclopropane-1-carboxylate oxidase 1, putative, expressed |
|  |  |  | *LOC_Os09g27830* | OsPDIL2-3 protein disulfide isomerase PDIL2-3, expressed |
|  |  |  | *LOC_Os09g27900* | plastidic glucose transporter 1, putative, expressed |
|  |  |  | *LOC_Os09g27940* | aspartic proteinase nepenthesin-1 precursor, putative, expressed |
|  |  |  | *LOC_Os09g27980* | emp24/gp25L/p24 family protein, putative, expressed |
|  |  |  | *LOC_Os09g28110* | hydroxyproline-rich glycoprotein family protein, putative, expressed |
|  |  |  | *LOC_Os09g28150* | bifunctional monodehydroascorbate reductase and carbonic anhydrase/nectarin-3 precursor, putative, expressed |
|  |  |  | *LOC_Os09g28180* | D-mannose binding lectin family protein, expressed |
|  |  |  | *LOC_Os09g28200* | HSF-type DNA-binding domain containing protein, expressed |
|  |  |  | *LOC_Os09g28230* | gibberellin receptor GID1L2, putative, expressed |

**Supplementary Table 8.** Linkage disequilibrium (LD) block analysis around the peak SNPs of *qBS3* and *q△BM9*.

| **Locus** | **Peak SNP** | **MAF** | **LD block result** | **Implication** |
| --- | --- | --- | --- | --- |
| *qBS3* | chr03_28324322 | 0.051 | No LD block delimited (maximum pairwise *r*² = 0.39; no SNP with *r*² ≥ 0.8) | Candidate region could not be narrowed; all four genes in the interval retained for expression and haplotype analyses. |
| *q△BM9* | chr09_16911051 and chr09_17059241 | 0.076-0.078 | Single strong-LD block (*r*² ≥ 0.8) spanning chr9: 16,829,251-17,249,775 (~420 kb) | Block covers essentially the entire QTL interval; candidate region could not be narrowed; all 36 genes in the interval retained for expression and haplotype analyses. |

**Supplementary Table 9.** Detailed haplotype composition, SNP position, and number of accessions of candidate genes (*LOC_Os03g49570*, *LOC_Os03g49610*, *LOC_Os03g49710* and *LOC_Os03g49730*) in *qBS3* with BLUP.

| **Gene** | **SNP position** | **Haplotype 1 (allele, n)** | **Haplotype 2 (allele, n)** |
| --- | --- | --- | --- |
| *LOC_Os03g49570* | 28,233,432 | C (316) | T (51) |
| *LOC_Os03g49610* | 28,252,107 | T (309) | A (58) |
| *LOC_Os03g49710* | 28,302,954 | C (316) | A (51) |
| *LOC_Os03g49730* | 28,310,051 | T (309) | C (58) |

**Supplementary Table 10.** Detailed haplotype composition, SNP position, and number of accessions of candidate genes (*LOC_Os09g27830*, *LOC_Os09g27900*, *LOC_Os09g28110* and *LOC_Os09g28180*) in *q△BM9* with BLUP.

| **Gene** | **SNP position** | **Haplotype 1 (allele, n)** | **Haplotype 2 (allele, n)** |
| --- | --- | --- | --- |
| *LOC_Os09g27830* | 16,935,592 | C (332) | T (37) |
| *LOC_Os09g27900* | 16,964,543 | G (331) | T (38) |
| *LOC_Os09g28110* | 17,067,124 | C (326) | A (43) |
| *LOC_Os09g28180* | 17,092,925; 17,094,700 | G, A (350) | C, T (19) |

**Supplementary Table 11.** Haplotype comparisons for the candidate genes of *qBS3* and *q△BM9*, with multiple-testing-adjusted *p*-values and effect sizes.

| **QTL** | **Gene** | **Hap1 mean** | **Hap2 mean** | **Mean diff** | **Cohens *d*** | ***P* value** | **FDR *q*** | **Bonferroni *P*** |
| --- | --- | --- | --- | --- | --- | --- | --- | --- |
| *qBS3* | *LOC_Os03g49570* | 654.41 | 600.12 | 54.29 | 0.41 | 0.002020 | 0.0040 | 0.0081 |
|  | *LOC_Os03g49610* | 653.25 | 612.88 | 40.37 | 0.30 | 0.024030 | 0.0240 | 0.0961 |
|  | *LOC_Os03g49710* | 654.41 | 600.12 | 54.29 | 0.41 | 0.002020 | 0.0040 | 0.0081 |
|  | *LOC_Os03g49730* | 653.35 | 612.34 | 41.01 | 0.31 | 0.021680 | 0.0240 | 0.0867 |
| *q△BM9* | *LOC_Os09g27830* | 1175.33 | 914.57 | 260.75 | 0.63 | 0.000406 | 0.0014 | 0.0016 |
|  | *LOC_Os09g27900* | 1174.61 | 927.71 | 246.90 | 0.60 | 0.000713 | 0.0014 | 0.0029 |
|  | *LOC_Os09g28110* | 1172.82 | 970.01 | 202.81 | 0.49 | 0.002821 | 0.0038 | 0.0113 |

Means are BLUP values; mean diff. = Hap1 mean – Hap2 mean (BS in g·mm², △BM in g·cm). *P* value: Student’s t-test. FDR *q*: Benjamini–Hochberg adjustment within each QTL. Bonferroni *P*: *P* × 4 (four haplotype comparisons per QTL).

**Supplementary Table 12.** Haplotype-phenotype association of promoter variants in the candidate genes of *qBS3* and *q△BM9*.

| **Target QTL** | **Gene** | **Promoter SNPs** | **Haplotypes (n ≥ 10)** | **ANOVA *P*** |
| --- | --- | --- | --- | --- |
| *qBS3* | *LOC_Os03g49570* | 27 | 4 | 6.88 × 10⁻¹¹ |
|  | *LOC_Os03g49610* | 7 | 3 | 1.20 × 10⁻¹¹ |
|  | *LOC_Os03g49710* | 18 | 5 | 1.42 × 10⁻¹¹ |
|  | *LOC_Os03g49730* | 24 | 4 | 1.26 × 10⁻¹¹ |
| *q△BM9* | *LOC_Os09g27830* | 18 | 5 | 5.07 × 10⁻²⁰ |
|  | *LOC_Os09g27900* | 15 | 4 | 5.45 × 10⁻²¹ |
|  | *LOC_Os09g28110* | 35 | 4 | 3.32 × 10⁻²⁰ |

**Supplementary Table 13.** Linkage disequilibrium (*r²*) among the non-synonymous SNPs of the candidate genes within *qBS3* and *q△BM9*.

| **QTL** | **Gene A** | **SNP A** | **Gene B** | **SNP B** | ***r^2^*** |
| --- | --- | --- | --- | --- | --- |
| *qBS3* | *LOC_Os03g49570* | chr03_28233432 | *LOC_Os03g49610* | chr03_28252107 | 0.860 |
|  | *LOC_Os03g49570* | chr03_28233432 | *LOC_Os03g49710* | chr03_28302954 | 1.000 |
|  | *LOC_Os03g49570* | chr03_28233432 | *LOC_Os03g49730* | chr03_28310051 | 0.820 |
|  | *LOC_Os03g49610* | chr03_28252107 | *LOC_Os03g49710* | chr03_28302954 | 0.860 |
|  | *LOC_Os03g49610* | chr03_28252107 | *LOC_Os03g49730* | chr03_28310051 | 0.960 |
|  | *LOC_Os03g49710* | chr03_28302954 | *LOC_Os03g49730* | chr03_28310051 | 0.820 |
| *q△BM9* | *LOC_Os09g27830* | chr09_16935592 | *LOC_Os09g27900* | chr09_16964543 | 0.971 |
|  | *LOC_Os09g27830* | chr09_16935592 | *LOC_Os09g28110* | chr09_17067124 | 0.845 |
|  | *LOC_Os09g27900* | chr09_16964543 | *LOC_Os09g28110* | chr09_17067124 | 0.819 |

**Supplementary Table 14.** Linkage disequilibrium (*r*²) between the GWAS peak SNPs of *qBS3* and *q△BM9* and the non-synonymous SNPs of the corresponding candidate genes.

| **QTL** | **Peak SNP** | **Candidate gene** | **Candidate SNP** | ***r*^2^** |
| --- | --- | --- | --- | --- |
| *qBS3* | chr03_28324322 | *LOC_Os03g49570* | chr03_28233432 | 0.0033 |
|  |  | *LOC_Os03g49610* | chr03_28252107 | 0.0044 |
|  |  | *LOC_Os03g49710* | chr03_28302954 | 0.0033 |
|  |  | *LOC_Os03g49730* | chr03_28310051 | 0.0101 |
| *q△BM9* | chr09_16911051 | *LOC_Os09g27830* | chr09_16935592 | 0.0091 |
|  |  | *LOC_Os09g27900* | chr09_16964543 | 0.0094 |
|  |  | *LOC_Os09g28110* | chr09_17067124 | 0.0108 |
|  | chr09_17059241 | *LOC_Os09g27830* | chr09_16935592 | 0.0094 |
|  |  | *LOC_Os09g27900* | chr09_16964543 | 0.0097 |
|  |  | *LOC_Os09g28110* | chr09_17067124 | 0.0112 |

**Supplementary Table 15.** Sensitivity analysis of the two stable QTLs (*qBS3* and *q△BM9*) under a stringent minor allele frequency threshold (MAF ≥ 0.10).

| **QTL** | **Year** | **Model** | **SNP** | **MAF** | ***P* value** | **FDR** | **Effect** | **△R² (%)** | **Significant (FDR<0.05)** |
| --- | --- | --- | --- | --- | --- | --- | --- | --- | --- |
| *qBS3* | 2023 | MLM | chr03_28578956 | 0.1567568 | 6.32E-03 | 0.9527 | 53.954 | 0.0164 | FALSE |
|  |  | FarmCPU | chr03_27849001 | 0.2243243 | 4.25E-02 | 0.9973 | -32.227 |  | FALSE |
|  |  | Blink | chr03_27831788 | 0.3783784 | 1.68E-02 | 0.4838 |  |  | FALSE |
|  | 2024 | MLM | chr03_28351862 | 0.2405405 | 2.61E-02 | 0.9853 | -39.096 | 0.0117 | FALSE |
|  |  | FarmCPU | chr03_28351862 | 0.2405405 | 3.46E-02 | 0.9333 | -37.48 |  | FALSE |
|  |  | Blink | chr03_28351862 | 0.2405405 | 3.46E-02 | 0.9333 |  |  | FALSE |
|  | 2025 | MLM | chr03_28200710 | 0.1432432 | 1.36E-03 | 0.9219 | 68.902 | 0.0231 | FALSE |
|  |  | FarmCPU | chr03_28220887 | 0.1648649 | 1.04E-03 | 0.8849 | 68.568 |  | FALSE |
|  |  | Blink | chr03_28220887 | 0.1648649 | 1.04E-03 | 0.8849 |  |  | FALSE |
|  | BLUP | MLM | chr03_28578956 | 0.1567568 | 3.72E-04 | 0.9569 | 44.095 | 0.0258 | FALSE |
|  |  | FarmCPU | chr03_27824363 | 0.1351351 | 4.05E-03 | 0.9829 | 43.843 |  | FALSE |
|  |  | Blink | chr03_28200710 | 0.1432432 | 4.25E-04 | 0.3793 |  |  | FALSE |
| *q△BM9* | 2023 | MLM | chr09_17089341 | 0.1162162 | 3.15E-07 | 0.0978 | 599.529 | 0.0605 | FALSE |
|  |  | FarmCPU | chr09_17089341 | 0.1162162 | 9.07E-07 | 0.2798 | 324.101 |  | FALSE |
|  |  | Blink | chr09_16684613 | 0.1081081 | 1.69E-08 | 0.0087 |  |  | TRUE |
|  | 2024 | MLM | chr09_17089341 | 0.1162162 | 7.85E-07 | 0.0857 | 557.382 | 0.0611 | FALSE |
|  |  | FarmCPU | chr09_16917568 | 0.1135135 | 6.93E-08 | 0.0357 | 401.526 |  | TRUE |
|  |  | Blink | chr09_17223880 | 0.1027027 | 7.49E-08 | 0.1156 |  |  | FALSE |
|  | 2025 | MLM | chr09_16917568 | 0.1135135 | 7.77E-04 | 0.6674 | 380.481 | 0.027 | FALSE |
|  |  | FarmCPU | chr09_16791549 | 0.1405405 | 2.22E-02 | 0.9998 | 114.611 |  | FALSE |
|  |  | Blink | chr09_16917568 | 0.1135135 | 1.49E-04 | 0.2844 |  |  | FALSE |
|  | BLUP | MLM | chr09_16917568 | 0.1135135 | 1.29E-08 | 0.0040 | 416.277 | 0.0712 | TRUE |
|  |  | FarmCPU | chr09_17301386 | 0.1081081 | 1.31E-13 | 2.02E-07 | 220.218 |  | TRUE |
|  |  | Blink | chr09_16917568 | 0.1135135 | 1.85E-16 | 2.86E-10 |  |  | TRUE |
